# A Critical Review of Recent Literature on Metal Contents in E-Cigarette Aerosol

**DOI:** 10.3390/toxics10090510

**Published:** 2022-08-29

**Authors:** Sebastien Soulet, Roberto A. Sussman

**Affiliations:** 1 Ingesciences, 2 Chemin des Arestieux, 33610 Cestas, France; 2 Institute of Nuclear Sciences, National Autonomous University of Mexico, Mexico City 04510, Mexico

**Keywords:** e-cigarettes, vaping, aerosol emissions, puffing protocols, metals

## Abstract

The inhalation of metallic compounds in e-cigarette (EC) aerosol emissions presents legitimate concerns of potential harms for users. We provide a critical review of laboratory studies published after 2017 on metal contents in EC aerosol, focusing on the consistency between their experimental design, real life device usage and appropriate evaluation of exposure risks. All experiments reporting levels above toxicological markers for some metals (e.g., nickel, lead, copper, manganese) exhibited the following experimental flaws: (i) high powered sub-ohm tank devices tested by means of puffing protocols whose airflows and puff volumes are conceived and appropriate for low powered devices; this testing necessarily involves overheating conditions that favor the production of toxicants and generate aerosols that are likely repellent to human users; (ii) miscalculation of exposure levels from experimental outcomes; (iii) pods and tank devices acquired months and years before the experiments, so that corrosion effects cannot be ruled out; (iv) failure to disclose important information on the characteristics of pods and tank devices, on the experimental methodology and on the resulting outcomes, thus hindering the interpretation of results and the possibility of replication. In general, low powered devices tested without these shortcomings produced metal exposure levels well below strict reference toxicological markers. We believe this review provides useful guidelines for a more objective risk assessment of EC aerosol emissions and signals the necessity to upgrade current laboratory testing standards.

## 1. Introduction

There is a broad consensus that “vapers” (users of electronic cigarettes (ECs)) inhale substantially lower content of toxic and carcinogenic compounds in comparison with tobacco smoke [1,2,3] (see [4] for a diverging opinion). This fact has motivated large numbers of smokers to adopt “vaping” (usage of ECs) as a significantly less risky alternative to smoking within the framework of tobacco harm reduction.

However, vapers are still exposed to the inhalation of harmful or potentially harmful compounds (HPHCs), particularly carbonyls, nitrosamines, metallic compounds and possibly carbon monoxide (CO) and Reactive Oxygen Species (ROS). Detection of metals in the chemical analysis of e-cigarette emissions is not surprising, as metallic compounds are already present in e-liquids at trace levels [5,6] and e-cigarette parts are made of various metallic alloys. Given their high level of toxicity and carcinogenic effects [7,8], it is a public health priority to provide vapers and smokers with an accurate analysis and evaluation of the involved risks of inhaling metallic content in adopting EC usage.

There is an extensive literature of laboratory studies analyzing metallic contents of e-liquids and EC aerosol (see descriptive review of experimental methodology in [9]). We provide in the present paper a critical examination of the more recent body of this literature consisting of 12 articles published after 2017 [10,11,12,13,14,15,16,17,18,19,20,21]. We will not deal with (i) studies on metal contents only in e-liquids and (ii) articles published before 2017, as older studies tested devices that are now obsolete [22,23,24,25,26]. Our emphasis is to examine the compatibility between puffing protocols, realistic usage and risk evaluation through comparison with toxicological references.

Aerosol collection techniques in the revised literature are diverse and a variety of devices have been tested, chemical analysis mostly relies in Gas Chromatography and Mass Spectrometry. However, there is a common generic feature in this literature: EC aerosols are artificially generated by puffing machines through regimented experimental protocols based on the ISO 20768 standard with puffing parameters defined by the the Cooperation Centre for Scientific Research Relative to Tobacco (CORESTA) protocol recommended method 81 [27]. This standard, which emerged as a natural adaptation to early vaping “ciga-like” devices of the standards used for laboratory testing of tobacco cigarettes [28], is followed (exactly or roughly) by almost all current laboratory testing of vaping devices. We will denote as CORESTA-like the puffing protocols that approximate the CORESTA protocol.

The puffing parameters of the CORESTA and CORESTA-like protocols are appropriate for vaping devices whose airflows and puff volumes are close to those of cigarettes [29], namely, low powered devices such as second generation clearomizers, tank equipped starter kits or pods, used with the ‘Mouth to Lung’ (MTL) vaping style with coil resistances above 1 Ω and power outputs typically below 20–25 W. However, CORESTA and CORESTA-like protocols are completely inappropriate to test high powered tank devices with coil resistances below 1 Ω (sub-ohm devices) designed to operate with much larger airflows, puff volumes and power outputs, used for the ‘Direct to Lung’ (DTL) vaping style (see [30] for comprehensive discussion on the relation between airflow and coil resistance).

It is not surprising that some of the studies testing sub-ohm devices with CORESTA-like puffing protocols found high levels of various metal elements that can even surpass toxicological markers (see for example [11,12,16]), but even if these markers are not surpassed (as in [10,18,19]) the obtained metal levels represent unrealistic exposures. The problem with these studies is not only usage of airflows and puff volumes that fall short of those for which sub-ohm devices were designed for their real life usage in DTL vaping, but also because this inadequacy very likely leads (even at relatively low power) to overheating conditions (see Soulet et al. [31,32] and Floyd et al. [33]), which for sufficiently high power might lead also to a ‘dry puff’ with depleted e-liquid and the coil pyrolyzing the wick [34,35]. Overheating conditions that increase coil temperature are known to correlate with sharp increases of the abundance of carbonyls in aerosol emissions [36] (see also [34,35,37,38,39,40]).

A useful way to determine experimentally, for any given combination of device and e-liquid, the parameters that should lead to the emergence of overheating (thus distinguishing normal vs abnormal operation modes) is the optimal regime defined by a linear relation between the mass of vaporized e-liquid (MEV) and supplied power that holds in a specific power range, with an overheating regime taking place above this power range where this relation becomes non-linear. As shown by Soulet et al. [31,32] the above mentioned relation between MEV and power is connected with the thermodynamical efficiency of the vaporization of the e-liquid prior to the formation of the aerosol.

Since ECs are aimed at real life consumers, it is important to bear in mind the limitations of laboratory testing, as there is evidence that regimented puffing by itself might produce (pending on the device and the puffing protocol) an increase of coil [37] and mouthpiece [38] temperatures that could be uncomfortable to end users (see example in [37]), thus suggesting to bear into consideration the specifications recommended by the manufacturer design, as well as users’ sensorial experiences.

Evidently, consultation or cooperation with human vapers in the testing procedure should be very helpful to determine testing parameters (see a welcome develpment on this issue in [41]). However, as far as we are aware, none of the studies on metal content that we have revised have done so. Disregarding these issues can lead to misleading emission outcomes from an artificial aerosol that is too hot and most likely repellent to end users, while the vaping machines (which do not taste nor feel) continue operating. Risk assessments under these conditions are of little utility for the end user (even under correct trapping and analytic techniques).

The revised literature exhibits other experimental flaws besides inappropriate puffing protocols for sub-ohm devices. In some studies tested devices were acquired months or years before the experiments without providing information on storage conditions: [14,15,16,18], thus raising the possibility of metallic components subjected to corrosion or degradation (this was recognized in [14,15,18]). Actual exposure from experimental outcomes was miscalculated in [10,11,13,16,18]. Important information on the device characteristics, aerosol collection and experimental outcomes was omitted in [12,13,15,16,18], making it very difficult to understand and evaluate the relevance and scope of their results (and to replicate the experiments). In particular, it is impossible to rule out testing of defective devices and cartridges in [14,15,18] that would probably be repellent to human users.

Most of the revised articles reported significant health risks and recommendations of strict EC regulation on the grounds of their laboratory outcomes. However, our findings in this review suggests that such conclusions are questionable, not only because they emerge from experiments with the methodological flaws that we have commented, but because even under the best possible experimental conditions the regimented puffing of laboratory testing provides at best an approximate proxy of human exposure. In this context, it is interesting to remark that studies on metal biomarkers in urine and plasma [42,43,44] do not seem to indicate serious short term health risks for human vapers (who most likely inhaled vaping aerosol under normal conditions, as opposed to a machine generated aerosol).

Laboratory testing is very useful for developing quality control standards, product comparison and technological development, but its capacity to asses health risks is limited. At best, laboratory outcomes might provide a reasonable inference of potential health risks from users’ inhalation of HPHCs as long as the experimental design is appropriate and puffing parameters (puff duration, puff volume, airflow) are roughly consistent with those of real life usage of the tested devices (information that can be gathered from consumer reports or manufacturer specifications).

Our section by section plan is as follows. Section 2 provides a description of real life vaping: vaping styles in Section 2.1 MTL and DTL vaping and habits of vapers in natural settings in Section 2.2, with reference values of various toxicological markers given in Section 2.3 presents. In Section 3, we examine the physical processes associated with EC aerosol generation and puffing parameters, while in Section 4, we revise the outcomes of the reviewed studies, offering a detailed discussion on their comparison with toxicological markers and a critique of their experimental methodology. In Section 5, we provide a comprehensive discussion on the findings of the previous section. A critique of risk communication in the reviewed literature is given in Section 6, while our conclusions are stated in Section 7. We also provide a supplementary file to explain the conversion of aerosol condensate concentrations into mass per puff values.

## 2. Realistic Usage Conditions and Toxicological Markers

### 2.1. Vaping Styles

The so called “Mouth to Lung” (MTL) vaping style is the most frequent one among vapers and currently remains typical of initiating users, most of them ex-smokers or current smokers. It involves mouth cavity retention followed by lung inhalation, a puffing mechanics roughly similar to that of cigarette smoking, thus being well suited for the design of early generation vaping devices (cigalikes, clearomizer models) and currently it is practiced in pods and tank models used as starter kits.

The “Direct to Lung” (DTL) style that avoids the mouth retention of MTL is typically practiced by more experimented and younger vapers. It involves a much deeper inhalation than MTL, which translates into more intense puffing parameters: airflow rates of 200 mL/s, puff volumes of 500 mL (or even more [45]), as well as longer puff times, resulting in much larger mass of inhaled aerosol. As opposed to the MTL style, DTL style bears no resemblance to tobacco cigarette puffing (as opposed to vapers, smokers tend to avoid a DTL style because tobacco smoke is a strong irritant [46]). Evidently, the heating element of vaping devices appropriate for this puffing regime must be able to deliver much higher power (combined with lower electric resistance) to generate the needed larger aerosol mass for a usage characterized by larger airflows for its inhalation. CORESTA and CORESTA-like puffing protocols are completely inappropriate and totally unrelated to consumer usage of sub-ohm devices intended for DTL vaping. Unfortunately, there is still no recognized standardized protocol to test devices intended for DTL usage.

### 2.2. Puffing Habits of Vapers in Natural Settings

In order to place laboratory studies in their proper context, it is important to examine the available information on the immense individual and circadian time variability of real life vaping. The best estimation of typical vaping behavior follows from observational studies of vapers under natural conditions carried on for extended periods (see review up to 2017 in [47]). Table 1 displays the main puffing parameters of 5 of such observational studies with information on daily puff numbers.

As shown in Table 1, the studies by Robinson et al. [48,49], Dautzenberg and Bricard [50] and Kosmider [51] report around 156–225 average daily puffs numbers for first and second generation devices, which are today obsolete or of marginal usage and whose nicotine delivery was much less efficient than that of more modern devices. In contrast, average daily puff numbers are in the range 272–338 in the more recent study by Dawkins et al. [52] involving more experienced vapers using modern devices (second and third generation) in which they can modify power settings and nicotine levels.

In the follow up study by Cox et al. [53] (see also [54]) larger daily puff numbers (308–338) and puff duration occurred when experienced vapers were asked to vape with fixed power settings and variable nicotine concentration. For the combination of low nicotine concentration and controllable power settings in third generation devices average daily puff numbers are around 272–279. As expected, inter-puff lapses under natural conditions listed in Table 1 are longer than those of laboratory studies.

Putting together the information described above and the data summarized in Table 1 and bearing in mind that both closed and open systems are currently in use, we believe that it is reasonable to assume 250 daily puffs as a rough but representative average value for real life daily vaping. In the following sections, we will use this value of 250 daily puffs to evaluate a daily inhaled dose of each metal element reported in laboratory studies in terms of various concentrations that will be converted to ng per puff.

### 2.3. Toxicological References

As mentioned in the introduction, laboratory testing does not reproduce real life vaping, but if puffing parameters used to generate the aerosol are appropriate for the tested devices, outcomes from laboratory testing can serve as valuable approximate proxies of human vaping to evaluate potential health risks in comparison with toxicological reference values. We consider the following three toxicological references:

- PDE-ICH: The International Council for Harmonization of Technical Requirements for Pharmaceuticals for Human Use (ICH [55]) provides the Permissible Daily Exposure (PDE) to inhalational medication, as a reference to manufacturing quality AFNOR-XP-D90-300 part 3 standard (page 15 of [56]). The ICH-PDE is endorsed by The US Department of Health and Human Services.
- ATSDR-MRL: The Minimal Risk Level (MRL) defined by the Agency for Toxic Substances Disease Registry (ATSDR) [57] as a safety limit for the general population of continuous daily environmental air concentrations (in µg/$m^{3}$) that can be of daily, intermediate (14 to 365 days) or chronic (over 365 days) duration.
- REL or PEL NIOSH-REL: Recommended Exposure Limits (REL) or Permissible Exposure Level (PEL) of the National Institute for Occupational Safety and Health (NIOSH) [58]. These are exposure limits that should be protective of worker lifetime safety to hazardous substances or conditions in the workplace.

Available values of these references for each metal element are listed in Table 2. We give priority to the PDE-ICH values, as these are strict protective and applicable to the general population, as well as already specified as a daily exposure referring explicitly to inhalation of medicines. While ECs are not medication, it is still useful to evaluate them under pharmaceutical standards. The ATSDR-MRL is also strict and applicable to the general population, given as a concentration defined to encompass safe continuous environmental exposure. The REL-NIOSH and PEL-NIOSH specifically refer to workplace exposure in terms of time weighed averages (TWA) working shift in 40 h weekly journeys. For metals without PDE-ICH we consider the MRL-ATSDR evaluated for a volume of $20\;m^{3}$ of inhaled air of average adults engaged in moderated activity. If there is no PDE-ICH nor MRL-ATSDR, we will use the PEL-NIOSH for a volume of $20/3=6.67\;m^{3}$ of inhaled air during an 8 h work journey of average adults engaged in moderated activity.

For the comparison of toxicological references in Table 2 with detected metal content in laboratory studies we evaluate a potential daily exposure in µg by multiplying the ng/puff = 0.001 µg/puff values in Tables 4, 5, 8, 10 and 11 times 250 daily puffs for average vapers that arise from studies of vaping patterns in natural settings discussed in Section 2.2 (for the REL-NIOSH we assume 83 puffs, one third of 250 daily puffs).

## 3. Optimal Regime, Power Ranges and Airflows

Efficient operation of ECs requires specific ranges of supplied power, temperature, coil resistance, inhalation airflow and puff volume. In particular, an optimal performance requires an appropriate airflow to efficiently generate an aerosol by condensation of the vapor generated by the supplied power. As mentioned in the introduction, all revised laboratory studies that looked at metal content in the aerosol generated by high powered sub-ohm devices [10,11,12,13,16,18,19] failed to fulfill this basic efficiency condition by testing the devices under inappropriate puffing protocols, specially low airflows and puff volumes (which also lead to enhanced production of carbonyls [36]). We discuss below the physical principles behind this issue.

ECs use as a heating element a wire or a mesh to heat and vaporise an e-liquid. They function between two typical powers: minimal and maximal, representing physical limits between three functioning regimes that are characterized at a first level using the Mass of E-liquid Vaporised (MEV) or e-liquid consumption expressed in mg by puff [31]. Below the minimal power no e-liquid is vaporized (MEV = 0) and no aerosol is generated (under-heating Regime). Between the two powers, MEV increases linearly with respect to the supplied power. This linearity denotes an optimal regime energetically efficient process of vaporisation under almost thermodynamic equilibrium conditions (this linearity followed by a non-linear behavior at higher power can be observed in Figure 4 of Floyd et al. [33]).

It is well known [32,59] that airflow rate [40,60,61] and e-liquid composition influence the power limits that define the optimal regime. A pure propylene glycol (PG) liquid has closer limits than a pure glycerol (VG) one. Adding a low concentration of ethanol and/or water in an e-liquid with a fixed PG/VG ratio slightly modifies the values. Then, testing the devices at a high airflow rate increases the power range between minimal and maximal values that define the optimal regime. This experimental observation is specially important for high powered sub-ohm devices used for DTL vaping, as testing these devices at a low airflow significantly reduces the power range of the optimal regime, with the overheating regime appearing at lower wattage.

Besides its influence in setting up the functionality limits of the optimal regime, airflow rate is the basic cooling process (through forced convection) during aerosol formation. The mixture of a hot and a cold gas is a fast process during which an important energy transfer occurs between air and vapor until they reach an equilibrium. This mixture leads to the formation of a “particle” phase in the form of liquid droplets whose composition is very close to that of the e-liquid. In fact, the higher is the airflow compared to the vaporized flow, the lower is the temperature of the mixture. This is supported by empiric evidence: for fixed power an increase of airflow tends to decrease coil temperatures and total particulate mass [60,61] and (at least) keeps the production of toxic byproducts (carbonyls) stable [40].

The right airflow depends on the supplied power. Since powerful devices vaporize a large amount of e-liquid, a large airflow is needed for the cooling through forced convection of the vapor to facilitate aerosol generation by condensation. A small airflow operating a powerful device will not carry on cooling through forced convection efficiently, leaving the atomizer full of hot vapor. In laboratory experiments characterized by a regimented repetition of puffs, the atomizer keeps accumulating heat even without e-liquid depletion (dry hit), increasing the temperature of the whole device (by conduction). While the vaping machines can continue operating, a human user would find first a very hot aerosol to inhale and later a device too hot to handle and most likely a repellent taste. In either case, testing a device under these conditions is completely unrealistic and misleading.

Once supplied power exceeds the maximal value of the optimal regime the relation MEV vs power becomes non-linear, marking the outset of an overheating regime characterized by different physical conditions under which the devices operate. This was discussed in a recent publication [62], suggesting that boiling processes are dominant in the optimal regime, with maximal power linked to critical heat flux. Following this assumption, boiling in an optimal regime would be through bubbles formed on the wire (nucleate boiling) whereas in overheating conditions, the wire would be surrounded by a film of gas, with vaporization taking place on the liquid–gas interface. Their results illustrate that under an overheating regime above maximal power, wire temperature increases significantly and carbonyls (specially formaldehyde) are produced in higher quantities, whereas in the optimal regime relatively small (even negligible) quantities of aldehydes are produced. This is consistent with the known relation between supplied power and carbonyl production [34,35,36,39,40].

Production of high levels of HPHCs (including metals) in the aerosol emissions from sub-ohm high powered devices might occur even at relatively low power when these devices are laboratory tested with a low intensity airflow (such as CORESTA or CORESTA-like protocols). This should be connected to the fact that the power threshold marking the outset of the overheating regime is lower when tested under such airflows in comparison with testing them with an intense protocol that fits the DTL parameters [32,59]. This suggests that a wider power range of the optimal regime in real life usage for DTL vaping should produce lesser levels of HPHCs.

Finally, it is important to mention that, regarding the puffing parameters, a regimented puffing regime can produce by itself a gradual temperature increase in the various components of the devices, even if the applied airflow is consistent with the device characteristics and the vaping machines keep the testing under the optimal regime. This temperature increase has been experimentally tested at the mouthpiece [38] and at the coil [37] (by thermography). While temperature increases reported by these references might not be accurate, this increase is plausible because the inter-puff time might not be sufficiently long to allow for the device temperature to decay to its initial value after each puff in frequent puffing testing, and thus as frequent puffs accumulate (with same supplied power) the devices can become too hot to handle for human vapers (or could have a repellent taste for them), but puffing machines operate normally.

## 4. Laboratory Studies: Outcomes, Toxicological Evaluation and Methodological Critique

We review, in this section, 12 articles published after 2017 [10,11,12,13,14,15,16,17,18,19,20,21] and listed in Table 3. For further discussion and comments see Section 5. There is in this literature a significant variation in aerosol collecting techniques, with Inductively Coupled Plasma Mass Spectroscopy (ICP-MS)) the preferred analytic technique (see descriptive review in [9]).

As mentioned in the introduction, a common feature is aerosol generated by puffing parameters based on the CORESTA Recommended Method 81 [27] or with parameters that approach it (CORESTA-like). Typically laboratory studies assume puff duration 3–4 s, inter-puff lapse 30–60 s, flow rate below 20 mL/s (1 L/min) and puff volume below 70 mL.

### 4.1. The Olmedo-Zhao Group

A group of researchers, originally from the Johns Hopkins School of Public Health, have published since their first article in 2016 [63] a series of articles on metal content associated with ECs, in e-liquids [42,64], on biomarkers in urine and serum samples of vapers [44] and on non-metallic contents in emissions from high powered devices [65]. The study by Olmedo et al. [10] in 2018 was continued by two more studies in collaboration with Zhao in 2019 and 2022: [11,12] and a review [9]. We examine below these studies.

The experimental method of the three papers [10,11,12] is specified in the 2016 article [63] with slight modifications: aerosol is generated by puffing e-cigarettes by a peristaltic pump, collection is done by direct condensation into a system of pipettes and tubes into a glass flask. The analytical technique is ICP-MS and the puffing parameters are listed in Table 3. The same experimental methodology was followed in more recent papers [13,65]. Since in the three studies [10,11,12] aerosol analysis by ICP-MS is performed on a liquid sample diluted from a condensed liquid aerosol of specified volume range in mL, it is straightforward to transform the interquartile values of μg/kg = ng/g concentrations into a range of ng/puff values listed in Table 4 and Table 5 (tank models) and 8 (pods), obtained from estimating of the mass of vaporized aerosol from the collected and retained aerosol and from the puff numbers needed to obtain the condensed aerosol under their puffing protocol (see details in our supplementary file). Comparison with toxicological reference markers is displayed in Table 4, Table 6 and Table 9.

#### 4.1.1. Olmedo et al. [10]

**Emissions.** The authors tested 56 devices and their e-liquids collected from recruited vapers for analysis. Besides studying metal contents in aerosol emissions, they provide valuable results by comparing metal content in e-liquids in dispensers and in tanks, before and after aerosol generation. Outcomes of metal elements in units μg/kg = ng/g were obtained in terms of self reported usage classification: voltage ranges (<4.02, 4.02–4.2, >4.2 V), coil alloy (kanthal and stainless steel and frequency of coil replacement). Since the information contained in these classifications is too vague (given the lack of data on individual devices), the most useful values of metal element content in aerosol emission is given in their third interquartile values listed in their Table 2 (middle column, second number in parenthesis). With the information provided on their experimental procedures we transform their μg/kg = ng/g concentrations values into a range of values in ng/puff for each metal (see details in the supplementary file). The outcomes for each metal are listed in Table 4.

The authors also provide at the end of their discussion section (for comparison with tobacco cigarettes assuming a smoked cigarette to be equivalent to 15 puffs) a median and a range of values based on their average puff volumes of ng per 15 puffs for six important metals (As, Cr, Mn, Ni. Pb, and Zn) in the emissions of the tested devices. Dividing by 15 the values they provide yields in ng/puff the following ranges and median values: <0.067 (0.01), As; <2.0 (0.0057), Cr; <0.093 (0.0013), Mn; <7.33 (0.029), Ni; <1.8 (0.007), Pb; <4.4 (0.299), and Zn. Save for Zn, these ranges are of roughly the same magnitude as the values we estimated in Table 4, but we will not consider them any further as there is no information on which specific tests these values were taken.

**Toxicological evaluation.** Olmedo et al. [10] claimed that 50% or more of the samples for Cr, Mn, Ni, and Pb exceeded toxicological reference values. However, as shown by Farsalinos and Rodu [66], they miscalculated in their Equation (1) the daily intake of these metals, as their conversion of μg/kg concentrations from chemical analysis into air density concentrations in mg/$m^{3}$ (for comparison with the environmental ATSDR reference value) is mistaken (see our Section 5.4). They assume for their experimental airflow Q=1 L/min and t=4 s puff duration that for each puff the collected aerosol would dilute in an air volume $V_{\mathrm{air}}=Q\times t=66.67$ mL, which is their experimental puff volume. Their estimations representing overexposures by at least a factor 12, since in real life usage the aerosol dilutes in a tidal volume of about 800 mL (assuming MTL vaping), about 30% larger than the rest tidal volume of ∼500 mL (this is because the lungs require extra volume to generate suction [45]). However, as we explain in Section 5.4, it is necessary to bear in mind that vaping represents an intermittent exposure, thus special care to incorporate exposure times must be exerted when comparing inhaled concentrations in users (from aerosol condensate concentrations) with time weighed toxicological markers (such as ATSDR or NIOSH). We find it more useful to compute the total dose for each metal per puff. We estimated (see supplementary file) an absolute range for these doses displayed in Table 4 given the uncertainty in the puff numbers needed (30–50) to collect a volume of aerosol (0.2–0.5 mL).

As shown in Table 4, none of metal elements examined by the authors of [10] produce a daily exposure that surpass the toxicological reference values. The metal that most approaches these values in Table 2 is nickel (a fraction about 1.685/6≈1/3.5≈28% of the reference value). For nickel to reach the PDE of daily intake of 6 μg a vaper would have to do 875 daily puffs. While some vapers might do this amount of daily puffs, demographic evidence displayed in Table 1 shows that such puffing frequency is an extreme outlier. It might be argued that the MRL-ATSDR values in Table 2 for nickel should be used because they are more strict than the PDE. In this case, assuming 20 $m^{3}$ of daily inhaled air by average adults we have: 4 μg for the intermediate MRL (14–365 days of exposure) and 1.8 μg for the chronic MRL (over 365 days of exposure). However, the daily exposure of 1.685 μg, computed for 250 daily puffs, is still below these strict thresholds, though the intermediate one is more realistic, as the the chronic one is a valid comparative reference only if one assumes a daily exposure to vaping that lasts at least a full year, which would indicate an abnormally and extremely intensive form of vaping.

**Methodological critique.** The authors did not provide complete information and characteristics of the individual 56 devices that were analyzed: coil resistance, power settings and PG/VG mixtures in e-liquids constitute important information to assess their results. The authors examined metal outcomes in terms of three self declared voltage categories: <4.02, 4.02–4.2, >4.2 V. However, the lack of information on coil resistance and power makes it impossible to determine if the tested devices were sub-ohm or operated for resistances >1 Ω. This is important information (see discussion in [30]) because the puffing protocol used in this laboratory study is inappropriate for sub-ohm devices used for DTL vaping that requires much larger airflows and puff volumes. Some of the missing information was supplied by Zhao et al. [11] who explicitly mention that 18% of the devices tested by the authors were the same sub-ohm devices they tested. This information is useful to interpret their statistical data: looking at aerosol emissions in the middle column of their Table 2 and the low wattage values (<4.2 Volts) in their Table 5 reveals a skewed distribution with a large interquartile dispersion and medians much closer to the lowest bound (first interquartile) than to the upper bound (third interquartile). This skewed distribution suggests that the possible 18% minority of tested sub-ohm devices produced unrepresentative ranges in the third quartiles, hiding the likely fact that for most of the devices the concentrations were closer to the lower bound given by the first interquartile.

#### 4.1.2. Zhao et al., 2019 and 2022 (Sub-Ohm Devices)

**Emissions.** Zhao et al. [11] published a study in 2019 following the same aerosol collection technique as Olmedo et al. [10] (with slight modifications), testing two sub-ohm devices of recent manufacture: OD1: Istick 25 (Eleaf Electronics) with power range 0–85 W and OD2: Smok (Smoktech) with power range 6–220 W, both with sub-ohm coil resistances. These devices were tested at three power settings: 20, 40, 80 W for OD1 and 40, 120, 200 W for OD2.

The authors published a paper in 2022 [12] to examine the effects on metal element content in aerosol emissions from varying flavorings (fruity, tobacco and menthol), nicotine concentrations (0, 6 and 24 mg/mL) and puff duration (2 s, 4 s and 6 s), utilizing exactly the same devices and aerosol collection technique as the 2019 paper, with fixed power for each tank device: 40 W for OD1 and 120 W for OD2. However, their reported outcomes lump together OD1 and OD2 in a single category “OD”.

Since the 2019 paper of Zhao et al. [11] followed the same experimental methodology and used same units as Olmedo et al. [10], we proceed as we did with the data supplied by the latter authors (see a detailed account of this conversion of units in the supplementary file). The range of ng/puff values we obtained for the sub-ohm devices tested by Zhao et al. in the 2019 study [11] appear in Table 5. We did not convert the metal elements in μg/kg = ng/g concentrations from their 2022 article [12] into ranges of ng/puff, since they did not provide in that study concentrations for individual devices, presenting only statistical data on concentrations corresponding to the various flavorings, nicotine concentrations and puff duration values lumping together the outcomes the devices OD1 and OD2 in the same category “OD”. However, their reported μg/kg = ng/g concentrations are qualitatively similar to those of their 2019 paper.

**Toxicological evaluation.** From the ng/puff values in Table 5 and considering an average of 250 daily puffs, we obtain daily exposure values for the open tank devices OD1 and OD2 for all metals and power ranges examined by Zhao et al. in their 2019 paper [11]. These daily exposure values only become comparable (or surpass) toxicological reference values listed in Table 2 for Cr, Cu, Mn, Ni and Pb and only in the highest power ranges of the devices. Daily exposure values for these metals and a comparative toxicological reference are listed in Table 6. For the pod devices CD1 (myblu) and CD2 (Juul), daily exposures are orders of magnitude below these references (see Table 8).

Zhao et al. [11] obtained from their Equation (1) and their μg/kg aerosol concentrations the following values for daily average exposure: 0.62μg (Mn) and 0.14μg (Ni), placed in their Table 4, but it is not clear how these values were obtained from their Equation (1), though they mention having followed the same exposure computation as Olmedo et al. [10], which (as we argued in Section 4.1.1) was shown to be incorrect by Farsalinos and Rodu [66] and might be conceptually problematic (see Section 5.4).

The values displayed in red in Table 6 correspond to daily exposure values of four metals (Cu, Mn, Ni, Pb) that surpass toxicological references by both devices in the high end of the power range of tests (80 to 200 W). Notice that for the device OD2 (SMOK) at its highest tested power (200 W), toxicological references are surpassed by 2 orders of magnitude in these metals. For the remaining metals daily exposure is at least an order of magnitude below toxicological references, even for iron and zinc which produced abundant content (but their available reference, the REL of NIOSH, is 1–2 orders of magnitude above). We do not offer a toxicological comparison of the outcomes of their 2022 paper because they lumped together data from both devices (OD1 and OD2).

**Methodological critique.** The 2019 study by Zhao et al. [11] is valuable for showing that all metal contents sharply increase with increasing supplied power (beyond manufacturers recommendations) while keeping the puffing parameters fixed but varying puff numbers. However, the authors’ assessment of health risks to end users by comparison with toxicological references is questionable. As we argue in Section 3, the excessively high outcomes reported by Zhao et al. [11] of Cu, Mn, Ni and Pb in their higher power settings (Table 5), with daily exposures surpassing toxicological references (Table 6), are linked to their testing of powerful sub-ohm devices (operating up to 200 W) by means of CORESTA-like puffing parameters (see Table 3) that fail short of the much larger values of the real life usage of these devices for DTL vaping (which is also the usage recommended by the manufacturers, in particular the manufacturer recommended power ranges of the OD2 device are between 20–50 W with best performance in the range 30–40 W [67]) (see Methodological critique in Section 4.7). Although lower power settings at 20–40 W of the sub-ohm devices are within the manufacturers recommended values and metal levels were below toxicological markers, the testing with inappropriate airflow and puff volumes render these outcomes unrealistic and likely overestimations with respect to real life usage.

The experimental design of Zhao et al. [11] required a large number of consecutive regimented puffs (120) to collect sufficient aerosol for the condensed 0.3–0.6 mL sample to be analyzed. Since the temperature of the heating element does not decay between puff to puff to the initial value, this long sequence of regimented puffs can easily produce a gradual heating of the atomizer to temperatures that gradually become too uncomfortable for the user to handle the device (besides the fact that users do not puff 120 regimented puffs every 30 s). This gradual temperature rise is a likely explanation of the large difference between the first and third quartiles in the concentrations $C_{i}$ for both sub-ohm devices in their lowest power settings (extreme left column in Table 2 of Zhao et al.): for example for nickel at 20 W in the Istik device there is a large interquartile range $\left(C_{\mathrm{Ni}}^{\left(1\right)},C_{\mathrm{Ni}}^{\left(3\right)}\right)=\left(5.89-222\right)$ ng/g, with median value ${\bar{C}}_{\mathrm{Ni}}=8.0$ ng/g, thus indicating a likely distribution of tests results clustered around the median value with large outlier values possibly at later puffs already with the device possibly too hot for a user to handle. The same phenomenon occurs for the Smok device at 40 W.

### 4.2. Zhao et al. (Pod Devices)

Zhao et al. also tested in their 2019 and 2022 papers [11,12] two pod “closed” devices: myblu (Imperial Brands) and Juul (Juul Labs), respectively, denoted CD1 and CD2, at their fixed power settings (the authors only identified CD1 as “BLU” but reading between lines it is evident that the device is a myblu). Separate outcomes for each one of the two devices were given only in [11], with both devices lumped together as “CD” in [12]. As we did with sub-ohm devices, we converted the μg/kg = ng/g interquartile concentrations they reported in Table 3 of their 2019 paper [11] for Cr, Cu, Ni, Pb, Sn, Zn into the ranges of ng/puff displayed in Table S8 (see the supplementary file). Considering the average of 250 daily puffs, the daily exposure for these two devices is 1–2 orders of magnitude below their corresponding reference toxicological marker, even for the relatively high concentrations values of Al and Cu.

It is interesting to consider nickel as an example. From the interquartile values in Table 3 of Zhao et al. we have the following ranges, for the myblu device $C_{\mathrm{Ni}}=1.32\;\left(0.39,3.35\right)\;\mathrm{ng}/g$ and for the Juul $C_{\mathrm{Ni}}=11.9\;\left(10.7,22.7\right)\;$. From these values, we obtain from Equations (3a) and (3b) of the supplementary file a nickel mass range of $M_{\mathrm{Ni}}=0.0016-0.056$ ng/puff for the myblu, while for the Juul we have $M_{\mathrm{Ni}}=0.014-0.066$ ng/puff. The range of daily nickel exposure (250 daily puffs) is then 0.0005–0.016 μg for the myblu and 0.0042–0.02 μg for the Juul, both ranges 2–4 orders of magnitude below the PDE of 6 μg for nickel. Notice that for the Juul device collecting the 0.3–0.6 mL of condensed aerosol sample required many puffs (290–330) taken at short inter-puff periods of 11 s. It is evident that even this small daily metal mass is likely an overestimation considering that such intense puffing regime is completely divorced from normal usage of this device.

In their 2022 study [12], Zhao et al. examined the effect of nicotine concentration and flavors on metal contents in emissions, but they report a joint outcome for CD1 and CD2 in a single category “CD”. This is problematic because each individual closed pod (besides operating at different powers) utilizes different type of nicotine in different concentrations: salts formed with benzoic acid (Juul, 59 mg/mL) and base (myblu, 24 mg/mL). Nicotine chemistry plays a role in the phase partition of the aerosol [68], with the less volatile protonated acidic nicotine (salts) tending to concentrate in the particulate phase and unprotonated (base) evaporating into the gas phase. While the implication of nicotine differences on metal content is not known, conflating both types of nicotine into a single statistic does not seem to be a correct approach.

### 4.3. Chen et al.

Chen et al. [17] conducted a comprehensive targeted study of chemicals in the emissions of the four Juul devices available in the US market in 2021: nicotine concentrations of 35 and 59 mg/mL in two favors: Virginia Tobacco (VT) and Menthol (Me), thus making four product combinations: VT5, VT3, Me5, Me3. The targeted analytes were divided in two groups (I and II) based on FDA USA guidance for vaping devices in its Pre Market Tobacco Authorization (PMTA) process. Each group was tested with different analytic methods, all validated according to ICH guidelines and standard ISO protocols. Depending on the analytic method aerosol collection method was by an impinger containing a trapping solvent or (for heavy metals) a glass fiber pad. Aerosol was collected for two puffing intensity regimes: “non-intense” (NI) with puff duration and inter-puff interval of 3 and 30 s, respectively, puff volumes 55 and 70 mL for group I and II, and “intense” (Int) with 6 s puff duration (the maximum allowed by Juul) and 110 mL puff volumes (other parameters unchanged).

Most of the analytes were below the limit of detection (BLOD) or below limit of quantification (BLOQ), though a thorough background subtraction was carried air blank measurements, with measurements for some analytes deemed not different from blank (NDFB) values. Six metals were targeted: Cd, Cr, Cu, Ni, Pb (group I) and Au (group II), with the numerical mass outcomes normalized with nicotine given for VT5 and Me5 in their Table 2 (quantifiable analytes) and averaging for the beginning, middle and end sequential puffing blocks we obtain the mass of these metals in ng per puff. These values are listed in Table 7.

As the authors comment, mass outcomes of these six metals are negligible and below BLOQ: Cd and Au were BLOD, chromium was NDFB and copper, nickel, and lead were alternately BLOD or BLOQ for all flavors, nicotine concentrations and puff blocks.

### 4.4. Liu et al.

The study by Liu et al. [13] specifically targeted arsenic species in e-liquids and in EC aerosol. The tested devices are not properly identified, only referred to as “*rechargeable USB-like devices … chosen based on their high market shares*” and “*tank type devices from two popular stores in Toronto, Canada*”. Aerosol collection resulted in 0.2–1 mL of aerosol condensate and 89–100% recovery, following the methods of the first 2016 paper by Olmedo et al. [63], with a button mechanism to activate the tank devices. The puffing topography was allegedly taken from [69] but the parameters do not correspond to that reference, but to the puffing parameters of the 2018 paper of Olmedo et al. [10]: 4 s and 30 s for puff duration and inter-puf interval, with airflow 1 L/min = 16.66 mL/s, using 40 puffs. The resulting arsenic species aerosol condensate concentrations in μg/kg are summarized in their Table 2.

Besides the lack of information on the devices and their characteristics and the problematic usage of a CORESTA-like puffing protocol for a sub-ohm tank device, Liu et al. [13] also incurred in the same miscalculation of Olmedo et al. [10] on the “air concentrations” in mg/$m^{3}$ to compare in their Section 2.3 with the occupational toxicological NIOSH marker (equivalent to the PEL OSHA) for arsenic and inorganic arsenic species in an 8 h work journey. As we comment in Section 4.1.1, Olmedo et al. [10] overestimated exposures by a factor of at least 12 (inhaled aerosol dilutes in a tidal volume of 800 mL for MTL vaping [45]), but also comparisons with time weighted toxicological references need to be carefully examined (see Section 5.4). However, even with this overestimation the detected concentrations found by Liu et al. [13] are below the PEL OSHA (same as NIOSH) of $10\;\mu {g/m}^{3}$. Assuming a user vaping with MTL style with tidal volume of 800 mL and correcting the overestimation by a factor of 12, the maximal reported value of arsenic concentration mentioned in [13] ($4\;\mu {g/m}^{3}$) becomes ∼0.33 $\mu {g/m}^{3}$, which is much smaller than the PEL NIOSH. This low value for arsenic species in EC aerosol is consistent with the fact that no other study looking at arsenic has found significant presence of this metal in aerosol emissions (for example, see for comparison ng/puff values in Table 5). As a consequence, the estimated cancer risk form arsenic inhalation evaluated in Section 2.4 of Liu et al. [13] is questionable.

### 4.5. Kapiamba et al.

The study by Kapiamba et al. published in 2022 [16] tested three devices, two low powered pod systems: a Juul (Juul Labs) and a Vapor4Life (XL pen EC, AUTO VAPOR ZEUS KIT, Vapor4Life Inc. Northbrook, IL, USA, ended sales in July 2021) and tank system VOOPOO (Drag X, Shenzhen Woody Vapes Technology Co., Shenzhen, China), all purchased in 2019. They do not use the standard CORESTA protocol, but the standard puff profile for tobacco cigarette aerosol measurements (ISO 3308:2000): 30 puffs with 2 s duration, 60 s inter-puff interval, 35 mL puff volumes and 1.05 lT/min = 16.67 mL/s. Aerosol collection through teflon filters and unspecified tubing. They conduct separate tests on aerosol metal contents to examine seven “tasks” (see Table 1 of [16]): (1) differences between devices, (2) flavors, (3) nicotine concentrations, (4) device power, (5) puff duration, (6) aging, as well as (7) environmental emissions through a respiratory model.

The article reveals a problematic lack of key information to understand its outcomes and several inconsistencies, for example:

- All devices were acquired in 2019, at least 2 years before the experiments and were possibly subjected to corrosion or leaching of metal alloys. The authors provide no information on their storage conditions.
- Their Table 1 states that zero nicotine and no flavor were assumed in tasks (1), (5) and (6), but these tasks involve a Juul and a Vapor4Life, devices that lack a zero nicotine option and are not flavorless (by “flavorless” we understand an e-liquid containing only solvents and possibly nicotine). It seems that the voopoo was tested with such an e-liquid, but the authors provide no information on the e-liquids used in its testing this tank device and the Vapor4Life.
- The authors provide in the abstract the following outcomes on ng per 10 puffs for chromium and nickel
  $$\begin{array}{cccc} \; & \mathrm{Juul} & \mathrm{Voopoo} & \mathrm{Vapor}4\mathrm{Life}\\ Cr & 117\pm 54 & 124\pm 77 & 33\pm 10\\ Ni & 50\pm 24 & 219\pm 203 & 27\pm 2 \end{array}$$
  which do not appear in the remaining of the article and there is no description in the abstract or in the body of the article on how they were obtained.
- In their Section 3, dealing with task (1), the only one involving the three devices, the authors report the following average ng per 10 puffs outcomes for nickel: 2.9±3.2 (Vapor4Life), 240.1±234.9 (voopoo), 50.3±24.9 (Juul), which are different from those given in the abstract. No explanation is given (were there different tests?).
- For the Juul device, the ng per 10 puffs range of values for chromium in the three favors of task (5): 73.24±44.2 (Menthol), 76.36±47 (Virginia Tobacco) and 107±83.5 (Classical Tobacco), significantly differ from the values for chromium in task (1) and with those mentioned in the abstract. This is strange because the unspecified Juul flavor in the test of task (1) should coincide with at least one of the flavor tests in task (5) and thus the outcomes should not differ much, as it should be the same testing protocol applied to the same device with same flavor. The authors provide no explanation on this difference.

The authors found high chromium levels for the Juul, comparable to those of the voopoo (a tank device). This is strange, not only because it is at odds with other laboratory studies [11,14,15,17], but because the Juul has an inbuilt control of the coil temperature that prevents operation under overheating conditions [17]. In addition, it is very odd that increasing supplied power (from 5 to 60 Watts) to the voopoo does not produce a significant increase in metal levels (as it clearly happens for example in [11]). It is possible that this odd outlier result emerges from corrosion effects in devices acquired 2 years before the experiments.

Kapiamba et al. also miscalculate their risk evaluation along the reasoning of Olmedo et al. [10] (see Section 4.1.1), but even in a more problematic manner. They assume a rest tidal volume of inhalation (450 mL) and compute the amount of breathed air in 10 puffs ($4.5\;\mathrm{LT}=4.5\times 10^{-3}m^{3}$), multiplying this quantity times the mg/$m^{3}$ concentrations of PEL of NIOSH for every metal, comparing this product with their ng per 10 puffs outcomes. However, as we argue in Section 5.4, this risk evaluation is conceptually mistaken, the PEL NIOSH is an occupational reference value obtained by time weight averaging of 8 h work shifts in 40 h week journeys, so it does not make any sense to compute it for the short time lapse of 10 puffs (besides the fact that PELs in general are higher for short term exposures). Kapiamba et al. also invoke (without providing a reference) the European Medicines Agency (EMA) to quote inhalation toxicological thresholds of 10 and 100 ng per day, respectively, for chromium and nickel. However, the EMA does not mention these values [55], it provides the PDE ICH of daily exposure for these metals that we have listed in Table 2 (3 and 6 μg for chromium and nickel, not 10 and 100 ng).

Contrary to the claims of Kapiamba et al., they did not examine environmental emissions (task (7)), but a sort of lung deposition model. Environmental emissions cannot be simulated by vaping machines because users retain a large percentage (∼90%) of the components of inhaled aerosol [70]. This is a confusing article, full of missing information and inconsistencies.

### 4.6. The CDC Group

Researchers from the CDC published two articles, the first one by Halstead et al. [14] and the follow up by Gray et al. [15], on metal contents in aerosol emissions following strictly the CORESTA 81 puffing protocol: 3 s puff duration, inter-puff lapse of 30 s, 55 mL puff volume and flow rate of 16.67 mL/s, using 75 puffs in [14] and 50 puffs in [15]. The experimental methodology (specially aerosol collection) and validation techniques are described in full detail in the fist paper: collection by fluoropolymer condensation trap built with high purity fluoropolymer to prevent metal leaching contaminating the samples, analytic analysis by ICP-MS. Using “spiked” e-liquids (i.e., inseminated with known metal content) they showed a very low rate of direct transfer of metal particles into the aerosol (between less than 1% to 4.7%).

The third paper by Pappas et al. [71] analyzed metallic particulate matter through single particle inductively coupled plasma–mass spectrometry (SP-ICP-MS) and dynamic light scattering (DLS), performing both single and dual element analyses to determine if particles are composed by individual or multiple metal oxides, with calibration and validation techniques that they describe in detail. Pappas et al. [71] tested the same type of devices as Gray et al. [15] and found similar anomalous outcomes as these authors did for elementary metal content. We discuss these results below.

**Emissions.** Halstead et al. [14] tested twelve devices, all acquired years before the experiments (2016–2018). The devices and acquisition date are: Vuse Menthol (2014 and 2017), Vuse Original (2014 and 2017), Njoy King Menthol (2016 and 2017), Blu Classic Tobacco single use (2014 and 2017), Logic Platinium (2014 and 2017), 21st Century Menthol, Regular, and Zero Nicotine (2014 and 2016), Joyeteck eGO tank device (2017), Juul (2018). They provide the outcomes of metal contents in their Table V as ng per 10 puffs, which we list as ng/puff for the Joyetech model in Table 11 and for the cartridge pods: Juul, blu and Vuse in Table 8 (together with pod devices examined by Zhao et al. [11]). We omit the values for the various cigalikes models that are no longer in use today (in fact, Vuse and blu devices acquired in 2017 are likely also discontinued).

The second paper by Gray et al. [15] tested three current usage pods acquired in 2019: Juul (Juul Labs), myblu (Imperial Brands) and Vuse Alto (R.J. Reynolds Vapor Company), with the following cartridge flavors: Mint and Classical Tobacco (Juul), (Intense Mint-sation and Tobacco Chill (myblu) and Menthol and Rich Tobacco (Vuse Alto). As with Halstead at al. [14], we report in Table 8 their outcomes (their Table II but in ng/puff) for seven metals (Cd, Cr, Cu, Ni, Pb, Sn, and Zn) for each device and flavor.

**Toxicological evaluation.** The devices tested by Halstead et al. [14] were all acquired well before the experiment: pods in 2017, the Juul in 2018 and the Joyeteck eGO in 2016 (though updated forms of the latter devices are still used). Even if there is a risk of corrosion (a possibility the authors acknowledge), it is evident from the ng/puff values listed in Table 8 that daily exposure is below toxicological references given in Table 2 for all metals they tested.

The second paper by Gray et al. [15] tested contents of same metals in aerosols of more recent cartridge pod devices: Juul, myblu and Vuse Alto, under the same experimental methodology as [14], each with tobacco-like and menthol-like flavors and high nicotine concentrations. The metal analysis found consistently low mass contents of all targeted metals in aerosol from the Juul devices, but surprisingly enormous variation of values were reported for the Vuse Alto device with Mint-sation cartridge (less in the tobacco flavor cartridge of the Vuse Alto and in both flavors of the myblu). It is not expected that cartridge based devices powered by 8 W can produce aerosol emissions with contents of Cu, Ni, Pb, Sn and Zn comparable to those of high powered sub-ohm devices tested by Zhao et al. in [11], but as shown in Table 9 this is what happens: copper content emitted by the Vuse Alto is higher than that of devices tested at 80–120 W (though it is still below the toxicological reference PDE of 30 μg in Table 2), while for nickel, lead and zinc the daily emission from the Vuse Alto are comparable to those emitted by the same sub-ohm devices tested at the same range 80–120 W, which surpass toxicological references.

**Methodological critique.** Halstead et al. [14] provide a valuable comprehensive discussion on trapping methods and validating techniques that were used in the follow up paper by Gray et al. [15]. They acknowledge the likelihood that their experimental outcomes have been affected by metal corrosion and degradation, as the devices were necessarily stored between 1 and 3 years before testing (most of them are no longer in use).

Gray et al. [15] also tested e-liquids from the pod cartridges, reporting specially high levels (in μg/g) of Cu, Sn and Ni in the myblu cartridges with flavor Intense Tobacco Chill (elevated but much lesser values were reported for Ni in the Vuse Alto cartridges of both flavors). As commented before, surprisingly high values also occurred in aerosol emissions only for one the Vuse Alto device with the flavor Mint-sation cartridge. These are outcomes restricted to a single combination of device and cartridge and thus require a proper explanation, as it is a clear signal of some special anomalous outlier situation affecting the tested cartridges, but not the pods, since significant lower outcomes occur with the same pod device and the other flavor cartridges. It is extremely unlikely that aerosol emissions from thousands of commercially sold Vuse Alto devices would exhibit, only for the Mint-sation flavor cartridges, such high metal levels (comparable to those of sub-ohm devices running at 80–120 W), without consumers having noticed this phenomenon likely in a foul testing aerosol (and consumer reports do note the existence of defective cartridges and pods).

Unfortunately, Gray et al. [15] provide very insufficient information on the tested devices and cartridges. It is impossible to know from the information they supply how many of the Mint-sation cartridges they tested produced such high metal outcomes (probably by being defective) or how large or representative is the sample they tested. This information should be accessible by placing it in a supplementary file, but the authors only provide minimal and maximal range of values in their test outcomes, not a median or average or any minimal descriptive statistics.

It would be very useful for consumers and regulators to know if the finding of high metal content in the Mint-sation cartridges was generic, as it would point out to a deficient quality control by manufacturers, but since the authors do not provide sufficient information on the samples, it is impossible to rule out that they acquired and tested a batch of unrepresentative defective cartridges. Another important information vacuum is on the precise test timing and conditions of storage in the 4 months time lapse they report between purchase and analysis of the devices and cartridges. They mention that the devices and cartridges had no manufacture or expiration dates, but this information can be supplied by the manufacturers. The authors do not report requesting such information and/or that it was denied. This lack of information hinders the understanding (and possibility of replication) of the authors’ results.

Although 4 months is a shorter period than the years between purchase and analysis in [14], it is a still a sufficiently large time to suspect a high likelihood of leaching and corrosion effects. While the authors do recognize this likelihood, they remark that it is an uncertain possibility and offer alternatively explanations deemed to be just as plausible: “pod-to-pod variability” or heating of internal components. However, we believe that such alternative explanations are very unlikely, given the large storage time and the fact that excessively high metal contents only appeared in one combination of pod and cartridges. The authors could have avoided this uncertainty (made more problematic by the lack of information) by involving end users in tasting the aerosol from pods with specific cartridges, as this would have signaled them whether the tested cartridges were defective or not.

The laboratory studies by Gray et al. [15] and Kapiamba et al. [16]were the only two among the 12 reviewed studies that found in low powered devices high levels of metal content in aerosol emissions (surpassing toxicological markers), though as we have argued above and in Section 4.5, neither one of these two studies supplied sufficient information to determine if these findings are representative of the products. Therefore, the authors’s conclusion in [15] that recent pod devices pose increasing health risks to users can hardly be sustained by their experimental outcomes.

The third study of the same group [71] by Pappas et al. estimated the number of nano-particles containing metallic oxides in the aerosol generated on (apparently) the same devices of Grey et al. [15] and resulting in analogous anomalies: consistently few particle numbers (less than 10,000) of all metallic oxides for the Juul device, higher but uneven numbers for both flavors of the mylu and tobacco flavor of the Vuse Alto, but extremely high number of particles of lead oxide (222,000) and huge variation for the Vuse Alto with tobacco flavor (nickel nano-particles per 10 puffs range between 630–190,000). As in [15], the authors do not provide a coherent explanation for these odd results, vaguely alluding to a high variability among devices and e-liquids, without any descriptive statistical analysis of samples (just ranges of values).

### 4.7. The Williams-Talbot Group

A number of studies has been undertaken by researchers of the University of California [18,22,23,24,72,73], providing useful assessments on the design of metallic parts and alloys in the coils, wires, solders and batteries of a large number of devices [22,72], the effects of aerosol collection techniques and puffing protocols the detected metal concentrations [18], as well as the evolution of these features with the introduction of newer devices [23,72].

**Experimental methods and exposures.** Three of the studies cited above [18,22,24] also obtained experimental results on metal contents in aerosol emissions, using either the CORESTA protocol or similar protocols and the analysis through induced coupled plasma optical emissions spectroscopy (ICP-OES) (the three papers refer their experimental methodology to [24]). We will not consider outcomes from earlier studies by this group [22,24] because the devices tested are no longer in use.

In a more recent study [18], the group tested several second generation cartomizer models: EgoC Twist mod with KangerTech Protank and Nautilus atomizers and iTaste MVP 2.0 with Kanger T3S atomizer (all acquired in 2014), a sub-ohm high power third generation kit model with commercial resistance (SMOK Alien) and two tank models Nemesis and iPV6X with reconstructed resistances (acquired in 2017). Their aims were to probe experimentally how two collection methods (impingers and cold trap) affect detected metal contents in aerosols emissions (the first laboratory study undertaking such comparison), to identify and quantify the transfer of metals into the aerosols produced by tank-style devices (they include cartomizers in this category), and to evaluate the effect of varying puffing topography. All devices were tested for “continuous” puffing (60 puffs of 4.3 s duration every 60 s) and “interval” puffing (clusters of 10 continuous puffs separated by 5 min brake).

Gathering all the information supplied by Williams et al. in [18] together with plausible assumptions based on the specifications of the devices manufacturers, we converted the μg/L concentrations into ng/puff values considering the maximal values for every metal reported in their supplementary files (see our supplementary file). These ng/puff values are listed in Table 10. Notice that silicon is abundant in all models dated 2014 (the three clearomizer models and the Nemesis Clone), something also reported in their previous paper [74] and likely related to wicks made of silica. It is worth remarking that the ng/puff values for their SMOK device are close to those reported by Zhao et al. in [11] for the tested open devices OD1 and OD2 in the 40 Watt power range (see Table 5).

**Toxicological evaluation.** In an early 2013 study [24] Williams et al. found silica and metal nano-particles and metal concentrations in the aerosol of cigalike devices. Farsalinos and colleagues [74] showed this metal content to be below occupational toxicological markers. In a 2015 study metal content in the aerosol of cigalikes and cartomizer devices was heavily dominated by silicon [22], likely generated from the silicon content of the wick/sheath of the tested devices or by leaching from the vessels of aerosol collection (see [18]), all other metals were detected in practically negligible concentrations. Since these studies looked at old devices that are now obsolete, we will not consider them any further.

Although Williams et al. did consider in their 2019 paper [18] combinations of various puffing parameters (“high/low” voltage HV/LV and flow rate HF/LF), these parameters do not deviate much from those of the CORESTA protocol and thus remain inappropriate for high powered devices used for DTL vaping. Still, for all metals and devices they tested the daily exposures are below PDE-ICH toxicological references. This can be easily appreciated by comparing the relevant toxicological reference in Table 2 with the product of each the ng/puff values in Table 10 times 250 daily puffs and converting to μg. In fact, the highest outcome in the study of Williams et al. is 14.44 ng/puff for nickel produced by their SMOK Alien device, leading to a daily exposure of 3.61 μg, which is below the PDE-ICH of 6 μg for nickel (it is even below the nickel intermediate MRL-ATSDR of 4 μg).

**Methodological critique.** The most innovative feature of the 2019 study by Williams et al. [18] is the experimental comparison of the effect of two aerosol collection methods, cold trap and impinger, on aerosol emissions, recommending the latter method for better performance (see further discussion in Section 5.6).

While the authors advice to minimize the amount of storage time before analysis, it is not evident that they followed this advice, since a major drawback of the study [18] is the fact that most devices were acquired in 2014, at least 4 years before the experiments, while the SMOK and iPVeX are dated at 2017. Unfortunately, the authors do not provide information on the storage of these devices and their parts. Another major drawback is testing devices with reconstructible resistances (RDA), as these are typically operated in very varied “do it yourself” manner, requiring constant wetting of the wick. In fact, it is not clear how did they machine puffed devices of this type and, evidently, such experiments cannot be reproduced.

Williams et al. [18] claim that concentrations of chromium, copper, lead, nickel, zinc in their own 2019 study exceed the OSHA PEL. As an example, they stress that the concentration of chromium from the tank-style device (Tsunami 2.4, a RDA model) reported in their supplementary file $5\times 10^{7}\;{\mathrm{ng}/m}^{3}$ far exceeds (by 4 orders of magnitude) $3.3\times 10^{3}\;{\mathrm{ng}/m}^{3}$, the OSHA PEL value for chromium. However, these comparisons are completely mistaken, as they are based on a mere comparison of concentrations from aerosol collection analyzed by an ICP-OES instrument and air concentrations disregarding the actual inhalation volumes. It is easy to prove this wrong. The chromium outcome that results from their Tsunami 2.4 device is 0.66–0.82 ng/puff (see Table 10), which multiplied times 250 daily puffs yields a daily exposure to chromium of 0.165–0.205 μg, which is between one and two orders of magnitude below the PDE ICH of 3 μg for chromium.

Both, Wiliams et al. [18] and Zhao et al. [11] al used the Istick 25 and a SMOK power units recommended for, respectively, 1–85 W and 6–220 W. For both devices they conducted the laboratory experiments outside these power ranges of best performance recommended by the manufacturers (besides using puffing protocols that do not correspond to real life usage of the devices for DTL vaping). There is also a vacuum of information: the mere commercial brand names do not identify a unique atomizer among the range offered by the manufacturers. Since the resistance value and coil metal alloy are reported to be Kanthal with 0.2 Ω for Istick and Stainless Steel with 0.6 Ω for SMOK, an internet search reveals that the Istick brand could be the Istick Pico 25 atomizers from Eleaf that have a power unit with a maximal electrical power of 85 W. The HW-N/M2/N2 coils equipped with the Ello atomizer could have been used, with recommended power range between 40 and 90 W with the optimal power in the range 65–75 W according to tests by Eleaf factory. Regarding the SMOK device, the Alien Kit with TFV8 baby atomizer has a power unit that could reach 220 Watts, while the TFV8-Q2 coil is built with stainless steel and resistance 0.6 Ω. Its recommended operation range is 20–50 W with best performance in the range 30–40 W. Both atomizers are recommended for DTL vaping.

In [18] Wiliams et al. tested 5 atomizers reporting their commercial name: Kangertech Protank, Aspire Nautilus, Kangertech T3S, SMOK alien kit (TVF8 Baby atomiser), Clone RDA and Tsunami 2.4 RDA without any additional specification. Two of the devices are rebuildable dripping atomizers that (as mentioned before) require a personalized “do it yourself” handmade coil building and are not designed for the usage of typical vapers, but rather for experimented *aficionado* type of vaper in a framework based on many trial and error repetitions to find the right power set-up for a desired sensorial feeling during vaping. Additionally, these devices require manual wetting of the cotton wick following changing patterns of the user subjective perception.

Evidently, testing this type of specialized devices requires a detailed dedicated study that takes into account their peculiarities, in particular the extreme difficulty to introduce any standardized procedure. Testing this type of RDA devices is clearly out of place in a publication based on regimented puffing patterns (all this besides the fact that applied airflow rates do not correspond to realistic usage by being the same or below the ISO:20768 requirements or CORESTA method 81). These devices have low air resistance leading to an inhalation close to natural breathing. Reaching the required airflow to be applied needs a physical restriction to increase lung pressure (i.e., mouth closing). It is quite uncomfortable and is consequently not representative of real use.

### 4.8. Other Laboratory Studies Detecting Metal Content

#### 4.8.1. Kim et al.

The authors examined changes in cariogenic potential in tooth surfaces exposed to e-cigarette aerosols generated by a sub-ohm tank device (0.2 Ω) running at 40 W, with atomizers filled with e-liquids (80/20 PG/VG percent mixture) with sweet flavors and nicotine concentration of 10 mg/mL [19].

E-cigarettes were puffed by a Universal Electronic-Cigarette Testing Machine (UECTM) developed by the American Dental Association (ADA), using a commercial sub-ohm tank (Aspire Cleito: 0.2 O Kanthal coil with cotton wick). Aerosols were generated at a power setting of 3.14 V (total of 49.2 W based on $W=V^{2}/\Omega$) determined by the manufacturer’s manual (capable up to 55–70 W). Each atomizer was used for 750 puffs (approximately 5 days usage) and replaced thereafter, taking care to replace atomizers performing abnormally. As puffing topography the authors considered what they describe as “published physiological human e-cigarette puffing topography”: 50 mL puff volume in 4 s puff duration every 18 s, justifying these parameters by their reference [46] (Behar et al.). They defined 10 puffs as one vaping session and 150 puffs as one-day use.

However, the puffing protocol used by the authors was that used by Behar et al. to test cigalike devices, collecting aerosols by a syringe and unspecified tubes, a completely inappropriate experimental methodology for testing a sub-ohm device at 49 W. As a consequence, their outcomes on cariogenic potential in tooth surfaces does not apply to real life vapers using such device. Nevertheless, the metal concentrations detected by their ICP-OES instruments were listed in their Table 3 for Ca, Cu, Fe, Mn and Si, remaining metals were either non-targeted of below LOD, all of them are well below the Threshold Limit Value of the National Institute for Occupational Safety and Health (TLV-NIOSH). We transformed their mg/LT into ng/puff in Table 11.

#### 4.8.2. Beauval et al.

The authors [20] used various analytic techniques (gas chromatography, high and ultra performance liquid chromatography and inductively coupled plasma with mass spectrometry or ultraviolet flame ionization detection) in order to identify the main e-liquid and its vapor constituents (PG, VG, nicotine), as well as potentially harmful compounds, all of which were found at negligible low levels: trace elements, including metals (≤3.4 pg/mL puff), pesticides (below quantifiable levels LOQ), polycyclic aromatic hydrocarbons (≤4.1 pg/mL puff), carbonyls (≤2.11 ng/mL puff). As a comparison these compounds in cigarette smoke, respectively, appeared as 45.0, 8.7, 560.8 and 1540 (in the same units). The device tested was a second generation Lounge with resistance 2.8 Ω at 3.6 V (∼8 W). The e-liquids had 65% PG, 35% VG, with the rest made of several and no flavorings, with zero and 16 mg/ml nicotine levels. Aerosol was produced through the CORESTA protocol: 55 mL puff volume, 96 puffs of 3 s duration every 30 s. Blank collection was conducted for all experiments. Most metals in aerosol emissions were found below LOQ, quantified concentrations were found of Al, Co, Mn No, Pb, likely from contaminations as they were comparable to those of the blank samples. Only Cd, Cr and Sb were present in some aerosol collections up to 0.14, 2.3 and 0.47 pg/mL per puff (as a comparison, As, Cd, Pb and Ti were quantified in the 3R4F cigarette smoke from 1.02 pg/mL for Ti to 44.98 pg/mL per puff for Cd).

#### 4.8.3. Palazzolo et al.

These authors [21] used as aerosol collecting method mixed ester celullose membranes and scanned electron microscopy as analytic technique. They examined metal contents of a second generation eGO Twist device in comparison with cigarette smoke (their control state). All metal element contents were reported below toxicological references.

## 5. Discussion

The previous section presented an extensive—article by article—review of 12 studies on metal content in EC aerosol published after 2017. We provide in this section further discussion and a summary that is itemized by shortcomings shared by various articles and other features.

### 5.1. High Powered Sub-Ohm Devices

All studies testing high powered sub-ohm devices [10,11,12,13,16,18,19] (mostly used and recommended for DTL vaping) did so by means of CORESTA or CORESTA-like puffing protocols that are appropriate for low powered devices used for MTL vaping, but not for DTL vaping that requires much larger airflows and puff volumes. While Olmedo et al. [10] claimed that 5 metals (Cr, Mn, Ni, and Pb) produced exposures above toxicological markers, their computation of these exposures was mistaken (see Section 5.4), their outcomes lead to exposures to all metals below toxicological markers (see Table 4). Outcomes of Liu et al. [13] (arsenic species), Williams et al., 2019 [18] and Kim et al., 2018 [19] also produced exposures below toxicological markers for all metals (see Table 10 and Table 11). Exposures surpassed toxicological markers in three studies: Zhao et al., 2019 and 2022 [11,12] (nickel, copper, lead and manganese, see Table 6) and Kapiamba et al., 2022 [16] (nickel and chromium). As we have argued in Section 3, these high levels of metals occur under testing conditions most likely affected by overheating outside the optimal regime. However, this testing of sub-ohm devices is unrealistic by failing to achieve even a minimal approximation to the real life usage of the devices. It is thus of little relevance to end users.

### 5.2. Pod Devices

All metal contents in pod devices were detected in negligible quantities well below toxicological markers in three out of five studies: Zhao et al. [11,12], Halstead et al. [14] and Chen et al. [17], with metal outcomes in the latter study being below quantification limit. However, outcomes for copper, nickel and lead where surprisingly higher than these markers (comparable to those found by Zhao et al. in [11], see Table 9), but only in Mint-sation flavor cartridges of the Vuse Alto device examined by Grey et al., 2019 [15]. As we argued in Section 4.6, a device operating below 10 W producing comparable metal output as sub-ohm devices tested at 80 and 120 W is a strange outlier result that raises suspicion of a defective cartridge subjected to leaching or corrosion that could have been repellent to users, Unfortunately, the authors do not provide sufficient information on their tested samples to verify or rule out these possibilities. Kapiamba et al. [16] also found high metal levels in the two pods they tested (Juul and Vapor4Life), but these are not reliable outcomes given the numerous inconsistencies of their study (see Section 4.5).

### 5.3. Testing Old Devices: Corrosion

Some of the studies (Williams et al. [18], Halstead et al. [14] and Kapiamba et al. [16]) tested devices that were acquired years before their laboratory testing (4 months lapse in Gray et al. [15]). None of the authors describes storage conditions, but [14,15] do recognize the risk of corrosion in testing such devices. The aim of these studies was not looking at the effects of corrosion or metal degradation from the aging of the devices and all authors are employed in public institutions in the US, where new devices can be easily bought in vape shops, thus it is hard to understand why they tested aged devices stored so much time before their testing.

Halstead et al. [14] examined the concentrations of metals in cartridges and pods of old devices. In all cases the older cartridges showed higher metal levels, thus indicating that longer storage time makes corrosion and leaching extremely likely. The 4 months between purchase and analysis in the devices and cartridges tested by Gray et al. [15], together with finding very high metal levels only in a single combination of pod/cartridge (Vuse Alto flavor Mint-sation), clearly favors corrosion effects over the alternative explanations suggested by the authors (product variability, heating effects, PH of e-liquids).

It is possible that leaching and corrosion might be more prevalent in closed systems because their cartridges are more likely to undergo longer storage time between their manufacturing and usage. Open devices are not stored with e-liquid and the delay between purchase, e-liquid filling and its vaporization for usage is typically shorter (below one or two days), thus reducing the likelihood of leaching and corrosion. While long time stored cartridges can be valuable in laboratory experiments to understand leaching, corrosion and degradation phenomena, it is irrelevant for most users typically consuming these products within the next few days after their purchase (though lack of proper maintenance by users might also cause these problems).

### 5.4. Comparison with Toxicological References

Olmedo et al. [10] claimed that exposure from their experimental outcomes was above the MRL-ATSDR toxicological markers for Cr, Mn, Ni and Pb. Liu et al. [13] and Kapiamba et al. [16] made similar claims in comparison with the PEL-OSHA, while Williams et al. [18] claimed that chromium levels in a sub-ohm device were orders of magnitude above the PEL OSHA by erroneously comparing concentrations in aerosol condensate and those of this occupational marker.

We can easily identify two basic mistakes in these exposure estimations: First, Olmedo et al. [10] (and Liu et al. [13] following suite) assumed that the inhaled aerosol dilutes in a puff volume (66.67 mL) generated by vaping machines or pumps, when it actually dilutes in a much larger tidal volume of 800 mL [45] (a fact that was noticed by Farsalinos and Rodu [66], though these authors assumed a resting tidal volume of 500 mL). Second, vapers are only exposed to EC aerosol while vaping (not continuously), but puffs are intermittent events lasting few seconds each and adding up to a reduced time lapse in a day of inhalation. Assuming 250 puffs of 3 s duration leads to a total of 12.5 min during the 480 min of an 8 h working shift inhalation (if using the PEL-NIOSH) or 1440 min (if using a daily value), which amounts to (respectively) 2.6% and 0.9% of the total times of exposure. It is important to bear this in mind, since toxicological references markers (PDE-ICH, MRL ATSDR and PEL-NIOSH) have been conceived and obtained for very specific exposure timeframes (see Section 2.3).

Comparison of concentrations while disregarding exposure times can be misleading. To look at an extreme case, consider the most worrying metal level we have estimated in our revision of metal studies: 0.147 μg of nickel per puff for the OD2 device tested by Zhao et al. [11] at 200 Watts (see Table 5). Assuming a puff diluted in 800 mL of tidal volume (not the puff volume of 66.67 mL from the vaping machine considered by Olmedo et al. in [10]) this leads to a concentration of 184 $\mu {g/m}^{3}$ for a single puff. This concentration seems enormous compared with the occupational concentration of the PEL-NIOSH: $C_{\mathrm{NIOSH}}=15\;\;\mu {g/m}^{3}$. However, once we take into consideration vaping exposure times within the 8 h timeframe of the PEL-NIOSH and the highest seasonal nickel concentration in Mexico City (to choose an extreme value in a polluted urban area: $C_{\mathrm{air}}=0.01953\;\;\mu {g/m}^{3}$, see Table 4 of [75]), we obtain a concentration that is about one third of the PEL-NIOSH concentration ($C_{\mathrm{NIOSH}}$):

(1) $$C=\frac{\Delta t_{\mathrm{puff}}}{t_{\mathrm{tot}}}\;\times N_{p}\;\times C_{\mathrm{vap}}+\left(1-\frac{\Delta t_{\mathrm{puff}}}{t_{\mathrm{tot}}}\;\times N_{p}\right)\;\times C_{\mathrm{air}}=4.803\;\frac{\mu g}{m^{3}}<15\;\frac{\mu g}{m^{3}},$$

where we assumed equal time ($\Delta t_{\mathrm{puff}}=3$ s) and equal aerosol concentration ($C_{\mathrm{vap}}=184\;\mu {g/m}^{3}$) for each puff and even put the daily $N_{p}=250$ puffs in these 8 h ($=t_{\mathrm{tot}}$). This concentration is still way above the daily MRL-ATSDR value for nickel (0.2 $\mu {g/m}^{3}$ for the intermediate timeframe, see Table 2). Moreover, the MRL-ATSDR marker is expected to be much lower than the PEL-NIOSH, as it is a toxicological threshold for the general population subjected to continuous longer time environmental exposure [57]. It is obtained from (typically) extrapolating from animal models to humans a NOAEL (No Observed Adverse Effect Level), a more strict toxicological criterion than the PEL. The longer the exposure timeframe the lower the MRL-ATSDR threshold becomes and the exposure assumptions are also more strict. The PDE-ICH is also a much stricter threshold than the PEL-NIOSH, it is also based on a NOAEL and can be also computed for continuous long term dosing [55].

The exposure comparison can also be accomplished in terms of mass doses. Intake of air diluted aerosol for the PEL-NIOSH concentration ($6.67\;m^{3}$ for 8 h) leads to an upper limit of nickel intake of 100.05μg, which is 2.7 times larger than than the daily intake of 36.79 μg from the OD2 device (see Table 6) for 250 puffs taken in 8 h. However, as expected, daily exposure dose with the MRL-ATSDR leads (for $20\;m^{3}$ daily inhaled air) to 4μg of nickel intake which is much less than the daily intake of 36.70μg from the OD2 device at 200 W.

To avoid problematic comparisons between concentrations of environmental toxicological markers and air diluted aerosol (which are problematic to evaluate and exhibit huge individual and time/space variation), we have preferred to incorporate the discrete intermittent nature of the puffing time exposure of vaping by going directly to comparison of intake doses, that is, by estimating the inhaled mass of a given metal per puff (from the experimental outcomes) and multiplying it by our estimate of 250 daily puffs to get a daily dose to compare it with the daily values of the PDE-ICH or the MRL-ATSDR, using the PEL-NIOSH with 83 puffs in 8 h only when the other two references are unavailable.

It is important to emphasize that we are comparing experimental outcomes with very strict toxicological markers that are applicable to the general population. As we showed above, even for the most worryingly high measured nickel levels (the OD2 device at 200 W) these levels are below the PEL-NIOSH occupational marker, while as shown in the tables of Section 4 those outcomes that surpass the more strict toxicological markers (MRL-ATSDR and PDE-ICH) do not correspond to real life usage and/or exhibit methodological flaws and (extremely likely) overheating conditions. Nevertheless, as we argue in Section 6, the occupational PEL-NIOSH can also be an appropriate toxicological marker for vaping, as a voluntary activity that is not aimed at the general population, but at adult smokers.

### 5.5. Information Vacuum

Failure to provide sufficient information on the devices, puffing protocols and outcomes hinders the evaluation of the quality and utility of laboratory studies. Several of the revised studies omitted valuable information. Olmedo et al. [10] tested 56 tank devices, without providing a list of individual devices (something they could easily have done in their supplementary material). They classified the devices in terms of voltage ranges, coil alloy and frequency of coil replacement, but not in terms of their resistance, which makes it impossible to determine their power range, thus analyzing together (what could be) very different tank devices: powerful sub-ohm and low powered tank ones. Since this distinction is technically very important [30], failure to provide this information hinders the evaluation of their results, as coil resistance and power are the main factors behind the increase of metal content (specially nickel, copper and lead) in EC aerosol emissions, as it was shown in the continuing paper by Zhao et al. [11] (though the CORESTA-like protocol used by both papers is inappropriate for sub-ohm devices).

In their 2022 study [12], Zhao et al. used the same devices as in their 2019 paper [11], but lumped together into a single statistic the outcomes of the two sub-ohm devices operating at two distinct powers (40 and 80 W) and the two pod devices (Juul and myblue). At least for the pods, these conflalatted outcomes do not seem to be reliable because the Juul uses nicotine salts (59 mg/mL) and the myblu basic nicotine (24 mg/mL), a fact that must bear influence on the aerosol phase partition and on its emissions (see comments in Section 4.2).

Liu et al. [13] just identified the tested devices as “USB-like” pods and a tank model, without specifying their characteristic parameters. Kapiamba et al. [16] (among many other irregularities) did not specify the coil resistance of the tested tank model. Williams et al. [18] also failed to provide an accurate description of the devices they tested (including one with a reconstructible coil), some of whom were purchased as far back as 2014. Grey et al. [15] did identify the pod models they tested, but did not provide sufficient information to analyze their outcomes, as the latter were given only in terms of mass ranges (in ng per 10 puffs) without a minimal descriptive statistics to understand their distribution (with high likelihood to mix frequent and outlier values).

### 5.6. Aerosol Collection

A critical examination of aerosol collection methods is essential in the evaluation of emission studies, as element leaching from various materials and vessels: glassware and plasticware (in tubings), ceramic containers and glass and quartz fiber filters, is a potential source of contaminants that can affect the outcomes of metal elements detected in EC aerosol. This leaching can be quantified by suitable acid presoaking of vessels and it must be taken into consideration to avoid detecting metal outcomes that can be overestimations.

There is no standard method for EC aerosol collection, so the studies on metals we have reviewed have utilized different methods: pipette tips and narrow tubing ([10,11,13]), syringe and unspecified tubing ([19]), high purity fluoropolymer tubing ([14,15]), tubing with teflon filters ([16]), Millipore Mixed Cellulose Ester membrane ([21]), cold trap ([18]), quartz pad extracted with 10% high purity nitric acid ([17]) and impingers ([18,20]). However, only two of the studies discussed in detail the possible contamination of metal outcomes by the materials of the collection method they used: Williams et al. [18] and Halstead et al. [14].

The detailed experimental comparison in [18] between the cold trap and impinger methods shows that, on average, the cold trap method yields higher metal contents than the impinger, but metal outcomes in each method depend on specific metals: some metals are only detected by the impinger method, which the authors showed to be more effective in collecting heavy metals, while the cold trap method was better with alkali, earth metals and metalloids. Though, the efficiency between collection methods also depended on the devices and on puffing topography through mechanisms that are still uncertain. For better accuracy, the authors of [18] recommend the impinger method that best avoids leaching from contact with large surfaces of tubing, acid soaking glass surfaces for increasing times (in day lapses) and avoiding large time storage after collection to prevent leaching from storage vessels (though it is not clear that they followed this advice in their study, see Section 4.7).

The authors of [14] also discussed the possible contamination by leaching from trapping systems, recommending the avoidance of EC aerosol collection by low purity quartz material and glass fiber filters, as well as aerosol trapping by electrostatic precipitation in high purity, fused silica quartz tubes, the preferred aerosol trapping technique of mainstream cigarette smoke. This is consistent with the large variability of metal outcomes when trapping EC aerosol through quartz filters [76]. They suggest aerosol collection by means of high purity fluoropolymer tubing, with the tubes characteristics found by appropriate validating techniques.

It is possible that some of the reviewed studies might have reported overestimations of metal outcomes from contamination from aerosol collection methods and materials, though it is beyond the scope of the present review to verify this possibility.

### 5.7. Metal Biomarkers

As opposed to metal content from machine generated aerosol in a laboratory, metal biomarkers are measured on body fluids of human vapers, whom we can safely assume carried on with normal usage of their devices, meaning without overheating and repellent flavor (most likely within the optimal regime). Metal biomarkers are then a more direct indicator of health effects based systemic absorption of vaping emissions by actual human subjects (as opposed to artificially generated aerosols). Three studies on metal biomarkers [42,43,44] found no statistically significant difference between vapers and non-users, thus suggesting that inhaled metal content under normal vaping conditions does not seem be of concern at least for acute exposure.

### 5.8. Comparison with Tobacco Smoke

All reviewed studies provide some comparison of their experimental outcomes with content of same metals in tobacco smoke, as ECs are conceived as harm reduction products providing a safer alternative to tobacco cigarettes. Several of the studies emphasize that nickel appears in comparable or larger mass content as in tobacco smoke (see for example Palazzolo et al. [21]). However, this comparison must be carefully examined, since metals in tobacco smoke and EC aerosol originate from different processes and involve larger content for different metals: the usually most abundant ones in EC aerosol (nickel and zinc) are often found in practically negligible amounts in tobacco smoke, while the most abundant metals in tobacco smoke [77] are either found in minute amounts (cadmium) or not detected (mercury) in EC aerosol.

## 6. Assessment of the Risk Communication

Most of the reviewed metal studies ([10,11,12,13,15,16,17,18,19]) have reported alarmingly high risks of health hazards from their experimental outcomes, even if (as we have shown in Section 4 and Section 5) in most of these studies such outcomes are below the reference toxicological markers listed in Table 2 and all studies detecting such high metal levels exhibit serious methodological flaws. Further, most of the revised metal papers take their risk assessments to suggest policy recommendations for stricter EC regulation.

On the grounds of our findings in the present review, we believe we need to question this risk communication, as it is based on laboratory outcomes often obtained when vaping machines operate with inappropriate puffing protocols that disregard real life usage, as well as other methodological flaws that we have described in Section 4 and further discussed in Section 5. For the same reasons stated before, we believe we need to question the conclusions on health hazards from metal content in vaping emissions found in the reviews by Zhao et al. [9] and Gaur et al. [78], as well as in the cancer risk assessment by Fowles et al. [79], as they are based on considering large metal levels that were obtained in laboratory studies whose shortcomings we have reported.

We also criticize a form of risk communication that emphasizes the comparable or higher levels of metal content with respect to tobacco smoke as a signal of EC toxicity, disregarding the fact that metals form merely a tiny fraction of the set of toxic and carcinogenic compounds found in tobacco smoke, while they are among the few trace toxic byproducts found in EC aerosol. As an example of this risk miscommunication, a 2013 study by Williams et al. [24] remarked that nickel was detected in amounts 200 times those of tobacco smoke, though these concentrations in EC aerosol were already negligible and well below toxicological markers (see Farsalinos et al. [74]).

Some of the reviewed studies recognize that laboratory testing does not reproduce human vaping, attempting to provide real life connection to their outcomes to justify their health risks assessments. In their 2019 study, Zhao et al. [11] allude to a “sensitivity analysis” stating that their outcomes are not affected by increasing the puff numbers from those of a session to real life daily puff numbers (which they assume to be 120, arguing that they might be reporting an underestimation of actual risks). This reasoning is incorrect, since the disconnection from real life usage in sub-ohm device testing in [11] is not a matter of counting puff numbers and comparing them with the surveys listed in Table 1, but of inappropriate puff volumes and puffing airflow required by the optimal operation of powerful sub-ohm devices used for DTL vaping. Other revised studies [10,12,13,15,16] have incurred in similar mistakes.

We have compared experimental outcomes of metal content of the 12 revised studies with various reference toxicological markers for 14 metal elements, giving preference to the PDE-ICH, a strict safety threshold applicable to the general population as a maximal daily intake of impurities in inhaled medication [55]. We have also placed for reference another strict safety threshold applicable to the general population: the environmental MRL-ATSDR [57]. It is worth mentioning that in all cases the experimental outcomes that produced exposures surpassing these strict toxicological markers were plagued by methodological flaws: testing sub-ohm devices in extreme power ranges disconnected with real life vaping [12], failure to provide sufficient information on tested samples to rule out testing unrepresentative defective cartridges [15], as well as a number of shortcomings discussed in detail in Section 4 and Section 5. For devices tested under appropriate conditions (and even those under inappropriate conditions but not at maximal power) the experimental outcomes lead to exposures below these strict markers.

We also refereed to the occupational toxicological references: PEL-NIOSH or REL-OSHA (see Section 2.3), whose application as safety thresholds to vaping has been criticized for “not being sufficiently protective” to the general population, or as stated by Williams et al. [18] (when discussing Potential health effects of EC elements/metals) because they are not “ recreational” safety thresholds. In this context, it is interesting to see the critique by Hubbs et al. [80] to occupational safety thresholds and the response by Farsalinos et al. [81]. While we prioritize a stricter reference such as the PDE-ICH to be on the side of more stringent precaution and do recognize the limitations of occupational thresholds, we believe that Farsalinos et al. are right in responding to this criticism and arguing the case for using occupational markers: vaping is not recommended for the general population or vulnerable individuals (infants, pregnant women or individuals with ill health), but for voluntary usage by adult smokers aiming at significantly reducing their exposure to the toxicity of tobacco smoke, a usage condition that is not much different from voluntary occupational exposure. Since “recreational” safety thresholds for vaping do not exist, other existing toxicological markers (occupational, environmental and medicinal) are perfectly applicable under their own limitations, together with the inherent limitation of laboratory testing that is (at best) a proxy to assess human exposure.

Finally, perhaps the over precautionary approach often expressed on the safety of vaping, demanding that it must be determined only by the strictest possible protective standards, comes from its mistaken association with smoking, which does require such strict level of protection. However, EC aerosol emissions are chemically and physically distinct from tobacco smoke and thus require completely different (and risk proportionate) safety and regulatory evaluation standards.

## 7. Conclusions

We have provided in this review an extensive critical revision of 12 laboratory studies looking at metal element content in EC aerosols published after 2017 (see Section 4 and Section 5). Nine of these studies are authored by researchers from academic and government institutions in the US, one from China (Liu et al. [13]) and one from France (Beuval et al. [20]). Only one study (Chen et al. [17]) is industry funded.

Our review mostly focused on the outcomes of metal elements, their comparison with reference toxicological markers and a methodological critique based on self-consistency and compatibility between puffing protocols and the characteristics and real life of the tested devices and compatibility with absence of overheating conditions that do not (necessarily) involve a “dry hit” condition associated with e-liquid depletion. We argue that this compatibility can also be associated to an optimal regime that can be tested in the laboratory (see Soulet et al. [31,32] and Floyd et al. [33]). As with other technologies, different ECs are suitable for different consumers and modes of usage that determine specific parameter ranges. Testing EC emissions must be compatible with these requirements.

Since all the 12 revised studies on metal contents (and likely most laboratory studies on non-metallic content) have relied on CORESTA or CORESTA-like puffing protocols, incompatible with the large airflows and high power input of sub-ohm devices, it is not surprising that high levels of certain metals (nickel, lead, copper, manganese) were found, specially at highest device power, surpassing strict toxicological references applicable to the general population (PDE-ICH and MRL-ATSDR). However, even if metal levels did not surpass these toxicological references, these outcomes are not realistic for coming out of experiments whose protocols are incompatible with real life usage of the devices. As a contrast, metal levels in the emissions of low powered devices (mostly pods, starting kits and second generation devices) were well below the strict toxicological markers in all self consistent laboratory testing, an expected and consistent finding given the fact that CORESTA or CORESTA-like protocols are still appropriate for testing such devices. High metal levels above toxicological markers were found in low powered devices in [15,16], but these are not reliable outcomes because these two studies are plagued by methodological irregularities (see Section 4.5 and Section 4.6).

We emphasize once more that laboratory testing is valuable for product comparison, quality control and technological advancement, but it does not reproduce human vaping experience (even under the best experimental conditions, regimented puffing might involve uncomfortable or repellent sensations for human users). While laboratory testing under extreme conditions divorced from real life usage might be of theoretical and practical interest in itself, it is irrelevant to assess health risks in users. However, well conducted experiments (appropriate puffing protocols and operating within manufacturer recommendations) may be useful to assess approximately the potential of health risks. Evidently, the full information that defines the device characteristics and puffing parameters must be fully and explicitly supplied in the materials and methods sections or in the supplementary files of the studies to render them valuable for consumers, public health officials and regulators. Studies conducted outside of these consistency parameter limits must explicitly notify the readership that the testing involves abnormal usage conditions (likely involving overheating or corrosion).

Unfortunately, most of the revised studies did not provide full information on key physical parameters (coil resistance, full specification of the device, manufacturer recommendation on power/voltage ranges and their experimental outcomes). None of the 12 revised studies relied on human subjects to confirm that testing conditions would (at least) minimally relate to users’ sensorial experience. However, it would be very useful for researchers on vaping emissions to involve human vapers (as done in [41]) and consult the information provided by manufacturers of the devices, as well as information contained in vaping magazines containing consumer opinions and experiences on recommendation of power, voltage and resistance, as well as the appropriate vaping behavior. This information is very useful, not only for comprehending the parameters associated with a safe and pleasant usage, but also for concrete technical advice on the experimental design to undertake realistic testing of the devices, contributing to improve the standards of EC testing in a laboratory. By ignoring this data researchers run the risk of conducting unrealistic experiments whose outcome would be an aerosol that real life users could find too hot and repellent. Such laboratory studies do not contribute to a public health benefit to the end user.

Our findings in this review point out to the pressing necessity to upgrade current laboratory standards, created for early devices and clearly inappropriate for efficiently testing the wide diversity of presently available devices. An upgraded standard needs to comply with real life usage of the devices and manufacturer specifications, as demanded by the Tobacco Product Directive (TPD) [82] of the European Union. Besides considering the appropriate puffing protocols that accommodate the diversity consumer usage as best as possible (considering useful technical guidelines discussed in [30,31,83,84]), it must evaluate tasting and sensorial quality of the generated aerosol by incorporating end users into the experimental protocol. An upgraded standard would not only be helpful to avoid some of the shortcomings in the studies we reviewed, but would be highly beneficial to all stakeholders: consumers, regulators, health professionals, governments and the vaping and tobacco industries.

Emerging “fourth generation” disposable pod devices provide another interesting avenue for future research. Their ease of usage and maintenance, together with their inexpensive pricing, explain the increasing prevalence of these devices in the vapor market [85], with justified concern for their increasing popularity among teenagers [86,87]. While there is already research on their flavorings [88] and organic byproducts in their aerosol emissions [89], a proper analysis of metal content in these emissions requires a thorough examination of their coils, plastic and metallic parts (solders, wires). Further laboratory testing of these devices is essential to provide informed safety guidelines to consumers, health professionals and regulators.

As future work we also aim at replicating some of the reviewed studies to verify the existence of overheating, testing also the same devices under more realistic conditions, as well as the compliance with the parameters of the optimal regime defined by Soulet et al. [31,32]. We also aim at reviewing laboratory studies on non-metallic trace compounds: organic byproducts [65,90], carbon monoxide [40,91,92] and free radicals [93,94,95,96,97,98], whose presence in EC aerosol emissions is also dependent on increasing device power and coil temperature in analogous manner as with metals. We believe the present review contributes to improve testing standards that are consistent with normal device usage and essential to assess objectively the public health impact of vaping products.

## Figures and Tables

**Table 1:** Puffing topography under natural conditions. The table displays the main puffing parameters in 5 studies on vapers in natural conditions for extended periods. Numbers are averages with the symbol ± denoting standard deviation, the letters CL, 2G, 3G stand for closed, second generation (cartomizer) and third generation (tank) devices. In Dautzenberg and Bricard the symbols denote: single isolated puff (a), 2–5 clustered puffs (b), 5–15 clustered puffs (c) and more than 15 clustered puffs (d). In Dawkins et al.: low nicotine level fixed power (1), low nicotine level variable power (2), high nicotine level fixed power (3), high nicotine level variable power (4), with 6 mg/mL and 18 mg/mL for low and high nicotine level. Notice that puff numbers and e-liquid consumption increase with devices operating at fixed power and with low nicotine concentration.

	Robinson 2015 [48]	Robinson 2016 [49]	Kosmider 2018 [51]	Dautzenberg & Bricard 2015 [50]	Dawkins 2018 [52]
Device	CL	CL	2G	CL & 2G	60% 2G 40% 3G
Follow up	24 h	1 week	24 h	116 days	4 weeks
puffs/day	225±59	162±78 (14–275)	156.2±95.3	163±138 (1–1265)	(1) 338±161 (2) 308±135 (3) 279±127 (4) 272±128
puff duration (s)	3.5±1.8 (0.7–6.9)	2.0±0.6 (1–3)	3.0±1.2	(a) 4.57±2.24 (b) 4.07±1.94 (c) 3.73±1.77 (d) 3.20±1.61	(1) 4.46±1.22 (2) 3.81±1.11 (3) 3.61±0.97 (4) 3.91±1.44
inter-puff interval (s)	47.7±12.1 (10–150)		15.4±22.0	(a) >60 (b) 19.26±15.12 (c) 16.77±13.23 (d) 13.68±11.53	(1) 34.22±20.08 (2) 39.32±26.8 (3) 41.22±26.23 (4) 37.32±27.18
puff volume (mL)	133±90 (9–388)	65.4±24.8 (24–114)	73.9±51.5		
airflow (mL/s)	37±16 (23–102)	30.4±9.2 (19–60)	24.7±10.2		
e-liquid per day (mL)					6.19±3.74 4.63±2.13 5.79±3.63 4.79±2.35

**Table 2:** Toxicological References. The table displays the minimal recommended values to avoid noticeable harm. The daily values for the MRL-ATSDR and REL-NIOSH are, respectively, computed for 24 and 8 h. The asterisks denote short term exposures (* daily, ** 15 days) and chronic exposure *** (more than 360 days).

Metal	PDE ICH µg/day	ATSDR MRL µg/$m^{3}$	Daily Value µg	NIOSH REL mg/$m^{3}$	Daily Value mg
Aluminum (Al)				5	33.3
Arsenic (As)	2				
Cadmium (Cd)	3	0.03 *	0.6	0.005	0.03
Chromium (Cr)	3			0.5	3.3
Cobalt (Co)	3	0.1	2.0		
Copper (Cu)	30			1.0	6.7
Iron (Fe)				5.0	33.3
Manganese (Mn)		0.3 ***	6.0	1.0	6.7
Nickel(Ni)	6	0.2 ** 0.09 ***	4.0 1.8	0.015	0.1
Lead (Pb)	5			0.03	0.2
Antimony (Sb)	20	1.0	20		
Silicon (Si)				5.0	33.3
Tin (Sn)	60	300 *	6000	2.0	13.3
Zinc (Zn)				5.0	33.3

**Table 3:** Laboratory studies on metal content in aerosol emissions published after 2017. The puffing parameters appear in this order: puff duration, inter-puff interval, puff volume, airflow rate. All studies have used puffing flow rates and volumes similar to the CORESTA 81 protocol. Aerosol collection (see Section 5.6) and analytic techniques are summarized in the text. We do not consider studies before 2017 because they involve devices that are either obsolete or of marginal usage.

Study	Device	Puffing Parameters	Analytic
	and Properties		Technique
**Third Generation Tank Models**			
Zhao et al.,		4 s, 26 s, 66 mL, 16.67 mL/s	ICP-MS
2019 & 2022	Smok, 6–220 W, 0.6 Ω	15–120 puffs	
[11,12]	Istick, 0–85 W, 0.2 Ω	15–120 puffs	
Kapiamba et al.,	Voopoo, 5–60 W	2 s, 60 s, 35 mL, 16.67 mL/s	ICP-MS
2022 [16]	Unspecified	30 puffs	
	resistance		
Liu et al.,	Unspecified	4 s, 30 s, 66 mL, 16.67 mL/s	ICP-MS
2020 [13]	3rd Generation	Unspecified puff number	Arsenic
	Tank Model		Species
Williams et al.,	Smok Alien, sub-ohm	4.3 s, 60 s, 30.1 mL, 7 mL/s	ICP-OES
2019 [18]	iPV6X, Tsunami 2.4 RDA	60 puffs	
	+ Nemesis Clone RDA		
Olmedo et al.,	56 assorted tank devices	4 s, 30 s, 66 mL, 16.67 mL/s	ICP-MS
2018 [10]		30–50 puffs	
Halstead et al.,	Joyetech eGO	3 s, 30 s, 55 mL, 16.67 mL/s	ICP-MS
2019 [14]	2016 Model	50 puffs	
Kim et al.,	Aspire Cleito, 0.2 Ω	4 s, 18 s, 50 mL, ∼20 mL/s	GC-MS
2018 [19]	Kanthal coil, cotton wick	150 puffs	
**Pods**			
Kapiamba et al.,	Vapor4Life	2 s, 60 s, 35 mL, 16.67 mL/s	ICP-MS
2022 [16]		30 puffs	
	Juul	2 s, 60 s, 35 mL, 16.67 mL/s	ICP-MS
		30 puffs	
Chen et al.,	Juul (not intense)	4 s, 30 s, 55/70 mL, 16.67 mL/s	ICP-MS
2021 [17]		3 blocks of 100 puffs	
	Juul (intense)	6, 30 s, 110 mL, not specified	ICP-MS
		3 blocks of 100 puffs	
Zhao et al.,	myblu	4 s, 11 s, 66 mL, 16.67 mL/s	ICP-MS
2019 & 2022		50–100 puffs	
[11,12]	Juul	4 s, 11 s, 66 mL, 16.67 mL/s	ICP-MS
		290–330 puffs	
Grey et al.,	Juul	3 s, 30 s, 55 mL, 16.67 mL/s	ICP-MS
2020 [15]	myblu	50 puffs	
	Vuse Alto		
Halstead et al.,	Juul	3 s, 30 s, 55 mL, 16.67 mL/s	ICP-MS
2019 [14]	Blu	75 puffs	
	Vuse		
	Obsolete disposables		
**Second Generation**			
Beauval et al.,	Lounge	3 s, 30 s, 55 mL, 16.67 mL/s	various
2017 [20]		96 puffs	techniques
Palazzolo et al.,	eGO	5 s, 10 s, 6.67 mL/s	Scanned
2017 [21]		45 puffs	microscopy
Williams et al.,	EgoC Twist Protank	4.3 s, 60 s, 17–81 mL, 4–19 mL/s	ICP-OES
2019 [18]	EgoX Twist Nautilus	60 puffs: continuous	ICP-OES
	iTaste MVP Kanger	& 10 min clusters	ICP-OES

**Table 4:** First rows are outcomes of metal elements reported by Olmedo et al. [10] given as ng/puff values converted from their µg/kg concentrations (see supplementary file). The second rows are daily exposures form 250 daily puffs and third rows are toxicological reference markers from Table 2. Minimal values in the range of $O∼10^{-3}\;$µg are not displayed.

Metal	Al	Cd	Cr	Cu	Fe	Mn
ng/puff	0.07–0.52	<0.01	0.002–1.02	0.03–1.19	0.002–1.65	0.001–5.5
daily exp. (μg)	0.0175–0.13	<0.0025	<0.255	0.0075–0.298	<0.4125	<1.375
Tox. Ref. (μg)	33,300 NIOSH	3 PDE	3 PDE	30 PDE	33,300 NIOSH	6 ATSDR
**Metal**	**Ni**	**Pb**	**Sb**	**Sn**	**Zn**	
ng/puff	0.03–6.74	0.02–0.86	<0.45	0.01–0.45	1.28–18.88	
daily exp. (μg)	0.0075–1.685	<0.215	<0.1125	<0.1125	0.32–4.72	
Tox. Ref (μg)	6 PDE	5 PDE	20 PDE	60 PDE	33,300 NIOSH	

**Table 5:** Range of mass (in ng) per puff of each metal element for the sub-ohm tank devices OD1 and OD2 tested by Zhao et al. in their 2019 study [11] at three power levels (the numbers are rounded up to two decimals). These values were computed from the range of concentrations in µg/kg = ng/g reported in Table 2 of Zhao et al. and the information provided by Zhao et al. on aerosol collection (see Supplemental file).

M	OD1 20 W	OD1 40 W	OD1 80 W	OD2 40 W	OD2 120 W	OD2 200 W
Al	0.02–0.04	0.04–0.14	0.09–0.61	0.04–0.14	0.10–0.42	0.2–2.50
As	<$10^{-3}$	0.01–0.04	0.02–0.10	0.005–0.01	0.006–0.045	0.05–0.58
Cd	<$10^{-3}$	0.0003–0.03	0.004–0.028	<$10^{-2}$	<$10^{-2}$	0.02–0.14
Cr	<$10^{-3}$	0.01–0.06	0.04–0.18	0.001–0.24	0.14–0.80	0.006–3.06
Cu	0.02–0.51	0.32–5.64	3.72–13.84	2.85–12.51	4.21–22.27	18.14–184.01
Fe	0.015–0.03	0.45–2.43	0.07–1.96	0.01–5.45	1.31–2.99	0.09–20.77
Mn	0.0002–0.03	0.11–0.27	0.36–2.11	0.02–0.65	0.53–2.00	0.13–6.94
Ni	0.02–1.55	4.27–13.69	3.94–34.64	2.95–18.20	0.29–56.95	12.93–147.17
Pb	0.01–0.27	0.59–1.61	7.91–39.31	1.41–28.99	4.62–14.09	11.06–198.80
Sb	<$10^{-2}$	0.02–0.15	0.03–0.20	0.01–0.22	0.02–0.08	0.11–1.08
Sn	0.002–0.054	1.85–7.01	0.32–2.16	0.11–1.92	0.22–0.73	0.55–11.37
Zn	1.06–4.79	15.28–48.04	87.07–344.87	6.99–145.86	8.89–26.61	53.48–1510.26

**Table 6:** Comparison of daily exposure of those metals from sub-ohm devices tested by the 2019 article Zhao et al. [11] whose daily exposure (in μg) surpass toxicological reference values (displayed in red). The meaning of PDE and REL is explained in Table 2 and in the text of this section. Daily exposures for the remaining metals are below available toxicological reference, including zinc and iron whose contents are large. We assumed 250 as the average number of daily puffs for typical vapers to evaluate daily exposure to potential users.

Device	Cu	Mn	Ni	Pb
OD1 20	0.005–0.12	<$10^{-2}$	0.005–0.39	0.002–0.07
OD1 40	0.08–1.41	0.027–0.067	1.07–3.44	0.15–0.40
OD2 40	0.71–3.12	0.005–0.16	0.737–4.55	0.35–7.24
OD1 80	0.93–3.46	0.09–0.52	0.985–8.66	1.98-9.83
OD2 120	1.05–5.57	0.13–0.50	0.07–14.24	1.15–3.52
OD2 200	4.53–46.0	0.03–1.73	3.23–36.79	2.76–49.7
Reference	30 (PDE)	0.3 (MRL)	6 (PDE)	5 (PDE)

**Table 7:** Mass in ng per puff for Juul devices tested by Chen et al. [17], for 50 mg/mL nicotine concentration and Menthol and Virginia Tobacco flavors (Me5, VT5) and non-intense and intense regime (NI, Int). NDFB stands for Not Different From Blank.

Me5						
**Metal**	**Au**	**Cd**	**Cr**	**Cu**	**Ni**	**Pb**
NI	0.0123	0.009	NDFB	0.015	0.798	0.004
Int	0.022	0.08	NDFB	0.019	0.827	0.005
**VT5**						
**Metal**	**Au**	**Cd**	**Cr**	**Cu**	**Ni**	**Pb**
NI	0.0126	0.008	NDFB	0.245	0.698	0.036
Int	0.0156	0.005	NDFB	0.067	0.108	0.045

**Table 8:** Mass per puff for pods devices tested by the CDC group ([14,15]) and Zhao et al. [11]. The values displayed in red correspond to the testing of the Vuse Alto (V. Alto) and myblu devices with Menthol flavor. Notice that nickel, lead, manganese and zinc outputs per puff from these particular cartridges are comparable to those found in the highest power settings of sub-ohm devices tested by Zhao et al. [11] listed in Table 5, thus suggesting an anomalous situation.

Study	Device	Cr	Cu	Ni	Pb	Sn	Zn
Hals- tead, 2019 [14]	Juul	< LOD	< LOD	< LOD	< LOD	< LOD	< LOD
	V. Alto M	0.05–0.17	< LOD	0.44–0.48	< LOD	< LOD	< LOD
	V. Alto T	0.03	0.05–0.21	0.11–0.27	< LOD	< LOD	< LOD
Gray et al., 2020 [15]	V. Alto M	0.89–2.99	1.71–20.9	15.8–37.3	9.65–46.3	0.98–4.41	86.7–458.0
	V. Alto T	0.01–0.18	0.1–1.46	0.05–9.79	0.09–1.63	0.01–0.03	1.0–4.05
	myblu M	0.06–0.07	14.6–17.4	3.1–10.8	0.05–0.17	8.12–12.7	<1.0
	myblu T	<0.05	4.61–5.32	0.015–0.13	0.05–0.29	0.94–5.1	<1.0
	Juul M	<0.05	0.1–1.6	0.05–0.2	0.06–0.08	0.01–0.06	0.5–1.78
	Juul T	<0.05	0.02–0.36	0.05–0.28	<0.05	0.01–0.05	<1.0
Zhao et al., 2019 [11]	myblu	< 0.012	0.076–1.13	<0.06	0.015–0.26	<0.013	3.23–41.29
	Juul	<$10^{-2}$	<0.022	0.01–0.06	<$10^{-2}$	<$10^{-2}$	0.76–2.50

**Table 9:** Daily exposure (in μg) of the Vuse Alto and myblu Menthol favors examined by Gray et al. [15]. A comparison (higher levels in red) is offered with daily exposure from same metals tested by Zhao et al. [11] on high power sub-ohm devices. The daily exposure was computed assuming 250 daily puffs.

	Vuse Alto Menthol	myblu Menthol	OD1 80 W	OD2 120 W	OD2 200 W	Toxicological Reference
Cr	0.22–2.24	0.015–0.017	0.01–0.04	0.02–0.2	0.001–0.77	3 PDE
Cu	0.43–15.67	3.65–4.35	0.93–4.35	1.05–5.57	4.53–46.0	30 PDE
Ni	3.45–9.32	0.78–2.7	0.98–8.66	0.07–14.24	3.23–36.79	6 PDE
Pb	2.41–11.57	0.01–0.04	1.98–9.83	1.15–3.52	2.76–49.7	5 PDE
Sn	0.24–1.1	2.03–3.17	0.08–0.54	0.05–0.18	0.14–2.84	60 PDE
Zn	21.67–114.5	<0.25	21.76–82.2	2.22–6.65	13.37–377.5	33,000 REL

**Table 10:** Mass (ng) per puff for devices tested by Williams et al. in their 2019 study [18]. These values were obtained from the concentrations reported in their supplementary file (See unit conversion in our supplementary file). All metal levels are below toxicological markers given in Table 2.

Device	EgoC T Protank	EgoC T Nautilus	iTaste MVP Kanger	Nemesis Clone	iPV6X Tsunami	Smok Alien
Al	0.08–0.11	0.03–0.05	0.09–0.14	0.16–0.2		0.27–0.36
Bo	0.52–0.75	0.18–0.26			0.32–0.40	
Ca	3.84–5.49	5.82–8.32	5.66–8.08	18.5–23.12	22.5–28.12	
Cd		0.002–0.003	0.002–0.003	0.006–0.007		
Cr		0.01–0.02	0.007–0.01		0.66–0.82	0.48–0.64
Cu	1.05–1.50	1.13–1.62	1.4–2.0		0.10–0.12	1.02–1.36
Fe				2.9–3.62	7.40–9.25	4.65–6.20
Ka	1.49–2.13	1.22–1.75	0.80–1.14	2.36–2.95		
Mg	0.09–0.13	0.3–0.4	0.08–0.12	1.76–2.20	1.70–2.12	
Na	0.60–0.87	2.17–3.11		9.4-11.75		
Ni	0.14–0.20	0.03–0.04	0.2–0.3	0.04–0.05	0.64–0.80	10.83–14.44
Pb	5.79–8.27	2.67-3.81	7.43–11.33	0.12–0.15	0.64–0.8	1.65–2.20
Si	23.0–32.8	24.5–35.0	15.39–21.98	23.28–29.10	2.12–2.65	1.74–2.32
Sn	1.78–2.54	1.03–1.47	2,42–3.45	0.60–0.75	3.64–4.55	1.8–2.4
Zn	0.64–0.99	3.16–4.52	0.88–1.26	0.5–0.62	8.7–10.87	23.67–31.56

**Table 11:** Metal elements in other studies (outputs converted in ng/puff). Kim et al. [19] tested a third generation sub-ohm tank device, the rest tested second generation devices. The values for Beauval et al. [20] are in picograms.

	Halstead 2019	Kim 2018	Beauval 2017	Palazzolo 2017
	[14]	[19]	[20]	[21]
Al				35.55
As				1.11
Ca		81.8		
Cd			0.14±0.3 (pg)	0.97
Cr			3.4±0.6 (pg)	
Cu	0.747±0.67	2.2		0.42
Fe		1.02		
Mn		3.4		0.02
Ni	0.495±0.19			0.53
Pb	1.14±0.4			0.13
Sb			0.47±0.3 (pg)	
Si		33.3		
Sn	0.04±0.01			
Zn	3.34			3.77

## Data Availability

Not applicable.

## Supplementary Materials

The following supporting information can be downloaded at: https://www.mdpi.com/article/10.3390/toxics10090510/s1.

## Abbreviations

The following abbreviations and units are used in this manuscript:

EC	Electronic Cigarette
HPHC	Harmful and Potentially Harmful Compounds
CO	Carbon Monoxide
ROS	Reactive Oxygen Species
CORESTA	Cooperation Centre for Scientific Research Relative to Tobacco
MTL	Mouth to Lung
DTL	Direct to Lung
TPD	Tobacco Product Directive
MEV	Mass of E-liquid Vaporized
PDE-ICH	Permissible Daily Exposure (International Council for Harmonization of
	Technical Requirements for Pharmaceuticals for Human Use)
MRL-ATSDR	Minimal Risk Level (Agency for Toxic Substances Disease Registry)
PEL-NIOSH	Permissible Exposure Level (National Institute of Occupational Safety and Health)
PEL-OSHA	Permissible Exposure Level (Occupational Safety and Health Agency)
NOAEL	No Observed Adverse Effect Level
PG	Propylene glycol
VG	Vegetable glycerine (glycerol)
ICP-MS	Inductively Coupled Plasma Mass Spectroscopy
ICP-OES	Induced Coupled Plasma Optical Emissions Spectroscopy
FDA	Food and Drug Agency,
PMTA	Pre-Market Tobacco Autorization
BLOD	Below Detection Limit
BLOQ	Below Quantification Limit
NDFB	Not Different From Blanks
EMA	European Medicine Agency
ADA	American Dentist Association
pg	picogram
ng	nanogram
μg	microgram
mg	milligram
g	gram
mL	milliliter
L	Litter
cm	centimeter
m	meter
h	hour
min	minute
s	second
Ω	Ohm
W	Watt
V	Volt
kPa	kilopascal
