# Supplementary material for: A Critical Review of Recent Literature on Metal Contents in E-Cigarette Aerosol
Source: Toxics. 2022 Aug 29;10(9):510. doi: 10.3390/toxics10090510 (PMC9506048; doi:10.3390/toxics10090510)
Supplement: Supplementary file 1 [file toxics-10-00510-s001.zip › toxics-1826051-supplementary.pdf]

Supplementary file: computation of exposures

0.1 The Olmedo-Zhao group

Olmedo et al [1] and Zhao et al [2] report their results as concentrations for each metal in units μg/kg = ng/g that were obtained from the analysis (through ICP-MS) applied on liquid samples from aerosol directly condensed without dilution as described in [3], which clearly states that these ng/g concentrations refer to the ratio of analyte mass to sample mass. For the different tested devices both studies used different number of puffs that were needed (depending on the device power) to collect roughly the same volumes of condensed aerosol samples: 0.2-0.5 mL in [1] and 0.3-0.6 mL in [2].

Given the uncertainty in the reported parameters, we can only infer an approximated range of mass of the total collected aerosol from the sample volumes, 80 % aerosol recovery and assuming the density of the sample of condensed aerosol to be equal to the density of glycerol (1.4 times water density). Given the range in mL of the sample volume and a specified range of number of puffs N_p needed to obtain it, we estimate ranges of total mass for each metal in units ng/puff from a suitable reported first and third interquartile values $C_i^{(1)}$, $C_i^{(3)}$, in units μg/kg = ng/g $= 10^{-9}$ (parts per billion ppb). To this estimated mass we add a 20 % extra from the estimated amount of aerosol retained by the instruments estimated by Zhao et al [2]. These ng/puff values would be equivalent to the mass of the metal element just before it would be inhaled by an imaginary vaper (before becoming diluted in a tidal volume of air).

0.1.1 Olmedo et al [1]

These authors tested 56 open tank devices taken from recruited vapers. There is no detailed information on the individual devices: brand, resistance, power range and coil resistance. Their experimental methodology is described in [3] (a methodology followed by as Zhao et al [2]). They needed 30-50 puffs from the peristaltic pump to collected 0.2-0.5 mL of samples of condensed aerosol in each test, reporting results as μg/kg = ng/g concentrations.

We estimate the range of sample mass to be $m_s = 0.28 - 0.7$ mg. With this information we can obtain an estimate for each metal mass content M_i in ng per puff incorporating the full range of uncertainty in m_s, C_i and puff numbers from the relation

$$M_i = M_i^{\min} - M_i^{\max}, \tag{1}$$

where

$$M_i^{\min} = m_i^{\min} C_i^{(1)}, \quad M_i^{\max} = m_i^{\max} C_i^{(3)}, \tag{2}$$

$$m_i^{\min} = \min\left(\frac{\text{range of}\, m_s}{\text{range of}\, N_p}\right), \quad m_i^{\max} = \max\left(\frac{\text{range of}\, m_s}{\text{range of}\, N_p}\right). \tag{3}$$

with the range of mass M_i of each metal content "i" in ng per puff obtained by substitution of the values m_i, C_i taken from the outcomes in Table 2 of [1] into equation (1) and (2)-(3), leading to

$$M_i^{\min} = 0.0056\, C_i^{(1)}, \;\; M_i^{\max} = 0.023\, C_i^{(3)}, \tag{4}$$

where $C_i^{(1)}$, $C_i^{(3)}$ are the first and third interquartile concentrations in ng/g reported in Table 2 of [1]. To verify that this is a reasonable estimate for generic tank devices, we remark that

aerosol mass yield per puff varies between 6-12 mg for a tank device at 20 W with $1\,\Omega$ coil resistance in a CORESTA regime [4], while the mass yield can reach 25 mg per puff for tank systems operating at higher power [5, 6] also in a CORESTA regime. Assuming a range of mass yield per puff of 6-25 mg, then the puff numbers to obtain an aerosol mass of 280-700 mg roughly fits the 30-50 puffs we are assuming.

0.1.2 Zhao et al [2]: sub-ohm devices

We proceed as we did with the results of Olmedo et al [1] using their reported parameters. The two sub-ohm devices tested by these authors are an istick 25 (Eleaf Electronics) with power range 0-85 Watts (denoted as OD1) and a Smok (Smoktech) with power range 6-220 w (denoted as OD2), both with sub-ohm coil resistances. Each one of these devices was tested at three power values: OD1, $20, 40, 80$ W and OD2, $40, 120, 200$ W. The number of puffs were:

- OD1, Istick: 20, 40 and 80 W, using 15-120 puffs, with 120 puffs for 20 W and 15 puffs for 80 W. We will assume 60 puffs for the intermediate value 40 W.
- OD2, Smok: 40, 120 and 200 W using 25-120 puffs, with 120 puffs for 40 W and 25 puffs for 200 W. We will assume 60 puffs for the intermediate value 120 W.

Given the ranges of sample volume 0.3-0.6 mL, puff numbers (see above), 80 % aerosol recovery and assuming the sample density to be equal to e-liquid density (1.12 times water density), we obtain a range of mass of total puffed aerosol of $m_s = 0.42 - 0.84$ g. Considering this range of m_s and the first and third interquartile values $C_i^{(1)}$, $C_i^{(3)}$ for each metal "i" from Table 2 of [2] (in μg/kg = ng/g), we can obtain an estimate range for each metal mass content M_i in ng per puff that incorporates the full range of uncertainty in m_s, C_i. Substitution of these values together with the corresponding puff numbers in equations (1) and (2)-(3) leads for each device and power combination

$$M_i^{\text{min}} = 0.0035\, C_i^{(1)},\;\; M_i^{\text{max}} = 0.007\, C_i^{(3)} \quad \text{(OD1 20W, OD2 40W)}, \tag{5}$$
$$M_i^{\text{min}} = 0.007\, C_i^{(1)},\;\; M_i^{\text{max}} = 0.014\, C_i^{(3)} \quad \text{(OD1 40W, OD2 120W)}, \tag{6}$$
$$M_i^{\text{min}} = 0.0168\, C_i^{(1)},\;\; M_i^{\text{max}} = 0.0336\, C_i^{(3)} \quad \text{(OD1 80W)}, \tag{7}$$
$$M_i^{\text{min}} = 0.028\, C_i^{(1)},\;\; M_i^{\text{max}} = 0.056\, C_i^{(3)} \quad \text{(OD2 200W)}, \tag{8}$$

To estimate the daily mass of each metal that would be inhaled by hypothetical vapers, we need to multiply the masses per puff above by the average numbers of daily puffs of vapers from demographic studies, which we take as 250 (see Section 2.2 and Table 1)

0.1.3 Zhao et al [2]: Pod devices

The pod systems tested were a myblu (CD1) and a Juul (CD2), both tested at fixed power. The number of puffs to collect the 30-60 mL of condensed aerosol were 290-330 puffs (Juul) and 50-100 puffs (myblu). For each metal, the mass per puff follows from (1) with

$$M_i^{\text{min}} = 0.0042\, C_i^{(1)},\;\; M_i^{\text{max}} = 0.0168\, C_i^{(3)} \quad \text{(Blu)}, \tag{9}$$
$$M_i^{\text{min}} = 0.0013\, C_i^{(1)},\;\; M_i^{\text{max}} = 0.0029\, C_i^{(3)} \quad \text{(Juul)}, \tag{10}$$

where the concentrations $\bar{C}_i^{(1)}$, $C_i^{(3)}$ are the first and third interquartile concentrations depicted in Table 3 of [2].

0.1.4 Williams et al 2019 [7]

These authors provide in their supplementary files concentrations in μg/L of 17 metal elements obtained from the chemical analysis for all the tested devices and for the variations in airflow and aerosol collection. However, they do not provide information on the mass or volume of the collected aerosol, which for the cold trap method was diluted in a round flask of 0.5 L containing a solution of deionized water, nitric acid and hydrochloric acid, while for the impinger syringe method it was collected into two glass impingers. However, it is possible to obtain the mass (in ng) per puff from the reported concentrations in μg/L = ng/g, general specifications of the manufacturers of the devices and the information on the voltage they used. For the resistances we assume a range of values recommended by the manufacturers, for example, for the three claromizer models we assume the range 1.8 to 2.8 Ω, which for the testing ranges 3.8-5.0 V in Table 1 of [7] yields by Ohm's law a power range of 5-13 W. The Nemesis device was tested at 3.7 V, while it is not described in their Table 1 as sub-ohm, RDA's are typically used at sub-ohm values that widely vary among users, thus we assume for a 2014 device 0.5 Ω, leading to about 27 W. The SMOK Alien was tested at 5 V and its resistance is specified by the manufacturer as $0.4\,\Omega$, hence its power had to be around 60 W. Assuming the same resistance for the iPV6X, its testing between 2.8 and 3.6 V yields a power range 19-32 W.

Second generation clearomizer devices that fit the voltage, resistance and power ranges of the same type of devices tested in [7] were also tested by Gillman et al [5]. From Table 1 of this reference, it is safe to assume that third second generation devices tested in in [7] produced an aerosol mass of $7 - 10\,\mathrm{mg} = 0.007 - 0.001\,\mathrm{g}$ per puff. Gillman et al [5] and Soulet et al [4] also tested sub-ohm tank models that should fit the range of parameters of the two RDA models tested in [7] (the Nemesis and the iPV6X). At around 30 W and 4 V these devices should vaporize $20 - 25\,\mathrm{mg} = 0.02 - 0.025\,\mathrm{g}$. The Smok device at 60 W and 5 V should vaporize an aerosol mass of 40-45 mg, but it is uncertain how much of this aerosol mass was puffed at such a low intensity airflow of 7 mL/s, hence we assume the range of mass of $30 - 40\,\mathrm{mg} = 0.03 - 0.04\,\mathrm{g}$. Considering these aerosol mass ranges, a range of mass m (in ng) per puff value for each metal and each devices follows by multiplying the ng/g concentrations C times these numbers:

- Second generation devices: $m = C \times 0.007 - 0.001$
- Nemesis and iPV6X: $m = C \times 0.02 - 0.025$
- Smok: $m = C \times 0.03 - 0.04$

The ng/puff values for each metal in the tables of the supplementary file of [7] are listed in Table 10 of the main manuscript.

References

[1] Olmedo, P.; Goessler, W.; Tanda, S.; Grau-Perez, M.; Jarmul, S.; Aherrera, A.; Chen, R.; Hilpert, M.; Cohen, J.E.; Navas-Acien, A.; et al. Metal concentrations in e-cigarette liquid and aerosol samples: the contribution of metallic coils. *Environ. Health Perspect.* 2018, **126**, 027010

[2] Zhao, D.; Navas-Acien, A.; Ilievski, V.; Slavkovich, V.; Olmedo, P.; Adria-Mora, B.; Domingo-Relloso, A.; Aherrera, A.; Kleiman, N.J.; Rule, A.M.; et al. Metal concentrations

in electronic cigarette aerosol: Effect of open-system and closed-system devices and power settings. *Environ. Res.* 2019, **174**, 125134

[3] Olmedo, P.; Navas-Acien, A.; Hess, C.; Jarmul, S.; Rule, A. A direct method for e-cigarette aerosol sample collection. *Environ. Res.* 2016, **149**, 151156

[4] Soulet, S.; Duquesne, M.; Toutain, J.; Pairaud, C.; Mercury, M. Impact of vaping regimens on electronic cigarette efficiency. *Int. J. Environ. Res. Public Health* **2019**, *16*, 4753.

[5] Gillman, I.; Kistler, K.; Stewart, E.; Paolantonio, A. Effect of variable power levels on the yield of total aerosol mass and formation of aldehydes in e-cigarette aerosols. *Regul. Toxicol. Pharmacol.* 2016, **75**, 5865.

[6] Li, Y.; Burns, A.E.; Tran, L.N.; Abellar, K.A.; Poindexter, M.; Li, X.; Madl, A.K.; Pinkerton, K.E.; Nguyen, T.B. Impact of e-Liquid Composition, Coil Temperature, and Puff Topography on the Aerosol Chemistry of Electronic Cigarettes. *Chem. Res. Toxicol.* 2021, **34**, 16401654

[7] Williams, M.; Li, J.; Talbot, P. Effects of model, method of collection, and topography on chemical elements and metals in the aerosol of tank-style electronic cigarettes. *Scientific reports* 2019, **9**, 114

999
